# Establishing Methyl 9‐Undecenoate as a Renewable Platform Molecule From Local Plant Oils for Catalytic Access to C_12_‐Polymer Precursors

**DOI:** 10.1002/cssc.71234

**Published:** 2026-11-14

**Authors:** Jerome Hommes, Jan H. Behler, Hendrik S. Lapainis, Dieter Vogt, Thomas Seidensticker

**Affiliations:** ^1^ Department for Biochemical and Chemical Engineering Laboratory of Industrial Chemistry TU Dortmund University Dortmund Germany

**Keywords:** oleochemistry, renewable resources, sustainable chemistry, value chain

## Abstract

The transition towards renewable carbon sources is essential for sustainable polymer production. Plant‐oil‐derived oleochemicals represent promising feedstocks due to their availability and compatibility with existing industrial processes. However, efficient and scalable routes from locally available plant oils to renewable C_12_ building blocks for polyamides and polyesters remain limited. Herein, the synthesis of methyl 9‐undecenoate as a renewable platform chemical from plant‐oil‐derived methyl oleate is demonstrated, together with its conversion into a diverse portfolio of C_12_ polymer precursors. Methyl 9‐undecenoate was produced by cross‐metathesis of methyl oleate with crude 2‐butene using a ruthenium catalyst system tolerant towards impurities in technical feedstocks. Optimisation established robust conditions with conversions of up to 90% and selectivities of up to 93%. The process was successfully scaled from 20 to 300 mL without significant loss of performance, enabling the isolation of methyl 9‐undecenoate with a purity of up to 99.4%. Subsequent isomerising methoxycarbonylation and hydroformylation provided 1,12‐dimethyl dodecanedioate and methyl 12‐oxododecanoate as key intermediates. These intermediates were further converted into symmetric and non‐symmetric C_12_ polymer precursors, including diols, diamines, dicarboxylic acids, and amino‐, alcohol‐, and carboxylic acid‐functionalised esters. This approach establishes methyl 9‐undecenoate as a versatile renewable platform chemical for more sustainable polymer precursor production.

## Graphical abstract

Development of a value chain to a diverse portfolio of renewable C_12_ polymer precursors via the sustainable platform chemical methyl 9‐undecenoate. Cross‐metathesis of local plant oils with underutilised raffinate 2, followed by isomerising functionalisation reactions, provides versatile polymer precursors.

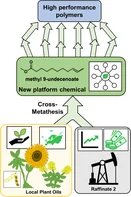

## 1 Introduction

The rapid implementation of bio‐based production routes in the chemical industry is most feasible when renewable molecules can directly replace established products within existing value chains. In such cases, already available infrastructure and transformation steps can be utilised, significantly lowering the barriers for industrial implementation [1, 2]. Particularly attractive targets in this context are intermediates for high‐performance polymers such as polyesters and polyamides, where slightly higher production costs can often be tolerated due to the high added value of the resulting materials. Additional advantages arise when underutilised feedstock streams and locally available raw materials are employed, thereby reducing dependence on imported resources and strengthening regional value chains [3, 4].

Plant‐oil‐derived oleochemicals represent promising renewable feedstocks for such approaches due to their wide availability and well‐established processing routes. In particular, methyl oleate obtained from local plant oils such as rapeseed, sunflower, or soybean oil constitutes an attractive starting material for the synthesis of value‐added intermediates. Transformations of long‐chain fatty acid derivatives already provide a wide range of industrial products including detergents, coatings, and lubricants [5, 6]. However, many polymer building blocks required for the production of polyesters or polyamides are still predominantly derived from fossil resources [7].

The general concept of converting plant‐oil‐derived feedstocks into renewable C_12_ polymer precursors is illustrated in Figure 1, together with potential applications and a comparison of different platform chemicals for their production.

**FIGURE 1:**
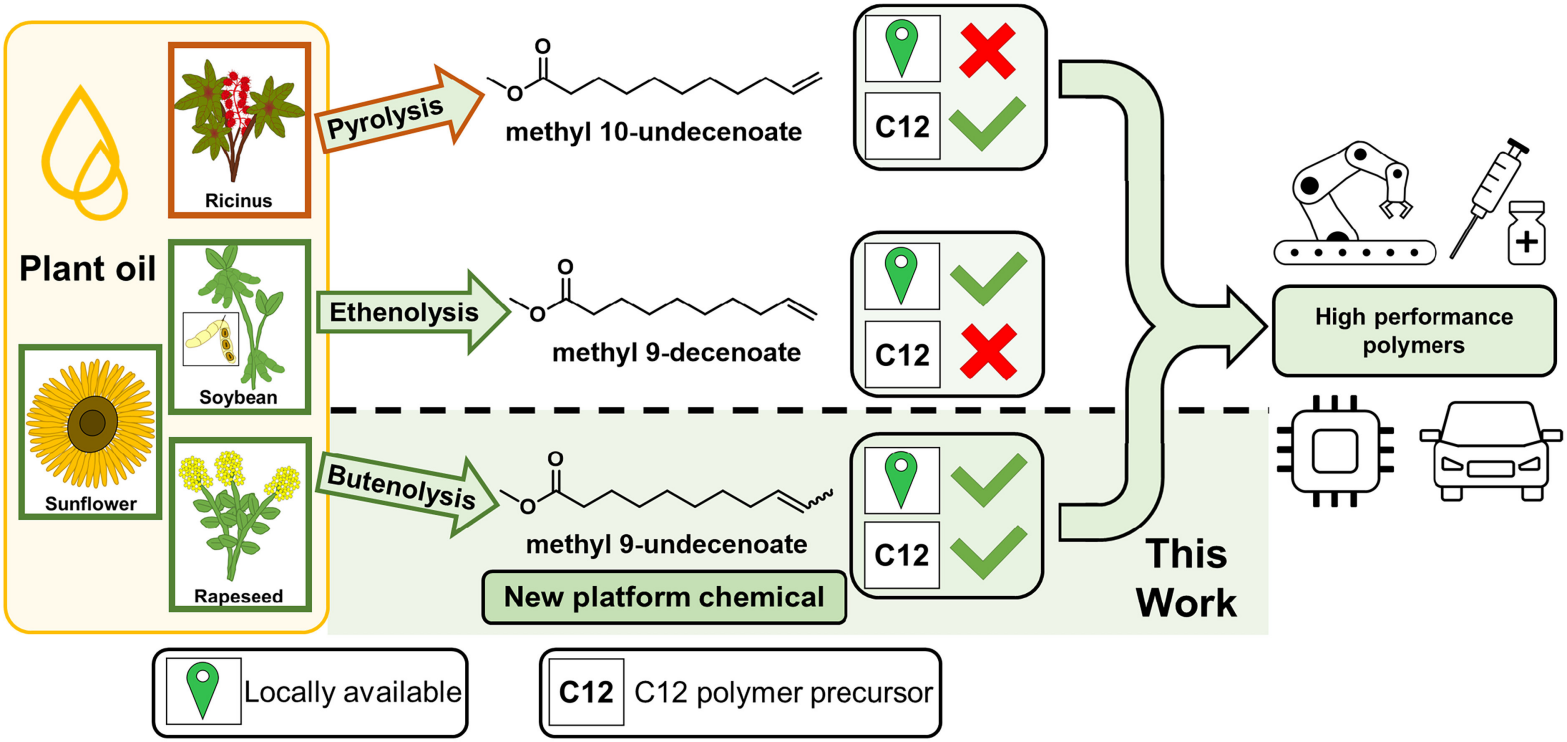
Comparison of platform chemicals derived from plant oils for accessing C_12_ polymer precursors.

Among these intermediates, C_12_ building blocks such as dicarboxylic acids, diamines, and other amino‐functionalised molecules are of particular interest as precursors for high‐performance polymer materials [8]. Developing sustainable routes towards such compounds from renewable feedstocks therefore represents an important objective in green and sustainable chemistry [9].

C_12_‐based monomers are particularly relevant for the synthesis of high‐performance polymers such as polyamide 12 (PA12) and polyamide 6,12. These materials combine excellent chemical resistance, mechanical stability, and low moisture uptake, making them highly attractive for demanding applications. Consequently, they are widely used in the automotive industry for fuel lines, in medical technology for catheters, in electronics as cable insulation materials, and in precision engineering for high‐performance components [10].

Although methyl 10‐undecenoate can provide access to related C_12_ polymer precursors, it is typically derived from castor oil, a feedstock that is not widely available from local sources in Europe. Another plant‐oil‐derived platform chemical, methyl 9‐decenoate, is limited by its shorter carbon chain, which prevents direct access to the targeted C_12_ polymer precursors. This limitation motivates the establishment of methyl 9‐undecenoate as an alternative renewable platform chemical derived from methyl oleate.

In this context, the present work investigates the synthesis of methyl 9‐undecenoate as a renewable platform chemical from plant‐oil‐derived methyl oleate and its subsequent conversion towards C_12_ polymer precursors. The key intermediate methyl 9‐undecenoate is synthesised via the cross‐metathesis of methyl oleate with 2‐butene. To facilitate industrial implementation and utilise inexpensive feedstock streams, raffinate 2, a crude 2‐butene mixture, is employed as the olefin source. Subsequent isomerising functionalisation reactions, including methoxycarbonylation and hydroformylation, provide access to the platform molecules 1,12‐dimethyl dodecanedioate and methyl 12‐oxododecanoate, which were subsequently converted into a range of C_12_ polymer precursors. A more detailed overview of the resulting value chain from plant oils to the corresponding C_12_ polymer precursors is presented in Figure 2.

**FIGURE 2:**
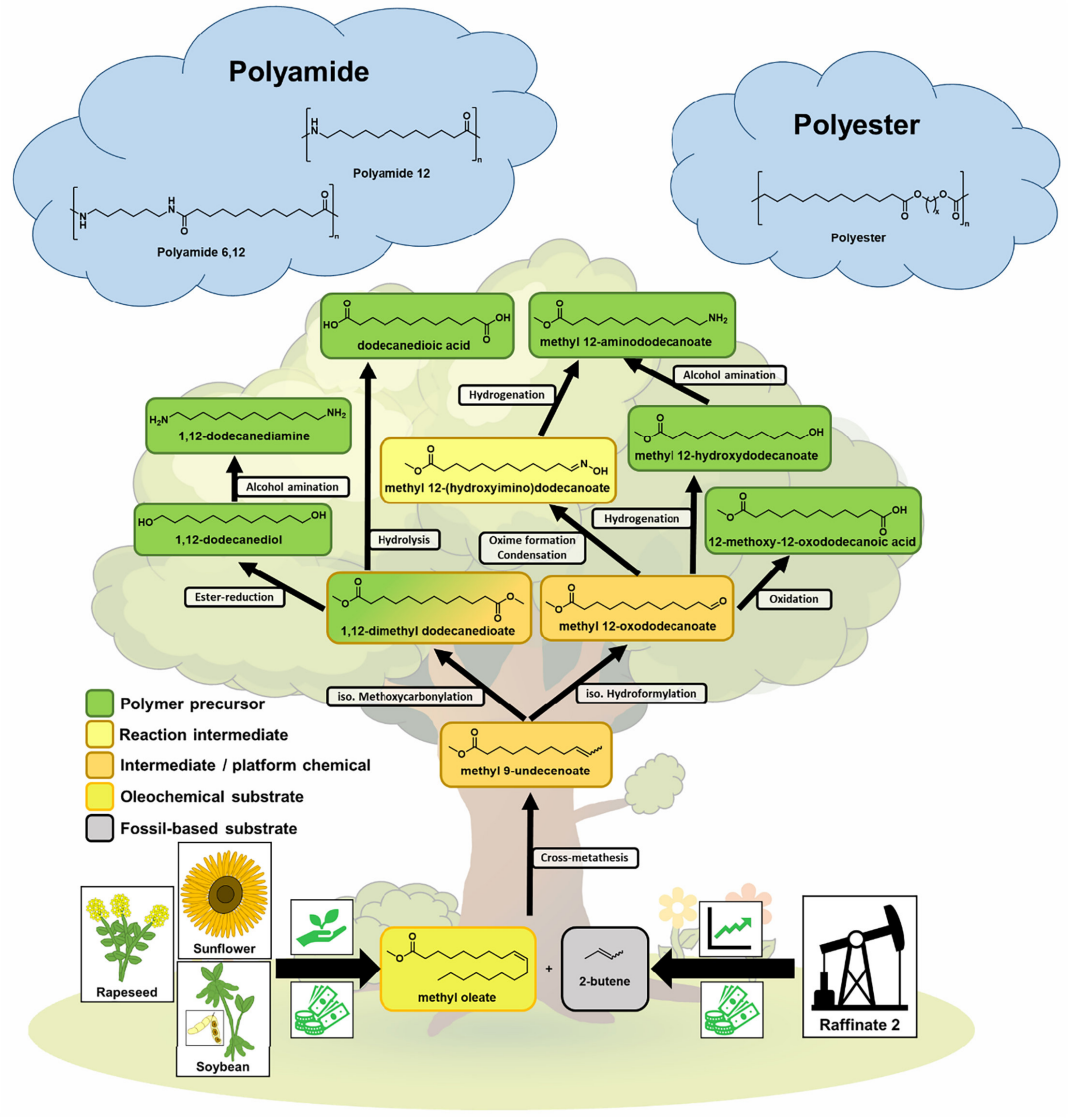
Polymer product tree based on methyl oleate. Possible polymer precursors formed from the cross‐metathesis product of methyl oleate with 2‐butene via isomerising methoxycarbonylation and hydroformylation reactions.

The aim of this study is to demonstrate that a complete value chain from local plant oils to C_12_ polymer precursors can be established with methyl 9‐undecenoate as the central renewable platform chemical using methyl oleate as the starting material. The main focus lies on the optimisation of the cross‐metathesis between methyl oleate and 2‐butene using a ruthenium catalyst, which exhibits high stability against impurities. Building upon the obtained methyl 9‐undecenoate, subsequent isomerising functionalisation reactions including methoxycarbonylation and hydroformylation are investigated, providing access to key intermediates that serve as versatile building blocks for further transformations towards C_12_ polymer precursors derived from renewable feedstocks.

## 2 Experimental

This experimental section describes only the standard procedure for the metathesis reaction. All other experimental procedures are provided in detail in the Supporting Information.

All reactions were carried out in reactors with volumes of 20, 75, or 300 mL. The 20 and 75 mL reactors were equipped with cross‐shaped magnetic stir bars to ensure efficient mixing, whereas the 300 mL reactor was stirred using an overhead stirrer. Both the 75 and 300 mL reactors were equipped with dropping funnels with pressure equalization.

Prior to use, all methyl oleate substrates were passed through a column of activated aluminium oxide to remove traces of water and peroxides. All Schlenk flasks used were heated under vacuum and subsequently purged with argon; this procedure was repeated three times. Methyl oleate was added to the Schlenk flask under argon counterflow and placed in an ultrasonic bath at 70 °C under vacuum for at least 30 min.

The catalyst, UltraCat, was added to the reactor under an argon atmosphere. Subsequently, the reactor was sealed, and methyl oleate was added under an argon counterflow.

The reactor was then connected to the 2‐butene mixture supply system, consisting of an inverted gas cylinder mounted on a frame placed on a scale, allowing precise monitoring of gas uptake. The entire apparatus was evacuated and subsequently purged with argon; this procedure was repeated three times until the system remained under vacuum, after which the filling process commenced.

The reactor was closed, and the connection between the reactor and the 2‐butene supply system was closed from the vacuum line. Subsequently, the valve of the 2‐butene mixture cylinder was opened, and the connection between reactor and 2‐butene supply system was filled with 2‐butene. After taring the scale, the valve connecting to the reactor was opened, allowing the 2‐butene mixture to flow into the reactor until the desired amount had been introduced. Following completion of the filling process, all valves were closed, and the line of the charging system was purged with argon. Subsequently, the reactor was disconnected from the filling setup.

The reaction began once the reactor was placed in its heating unit, where it was continuously stirred and heated.

For reactions conducted in 20 mL reactors, the reaction was stopped by cooling in an ice bath, followed by three purging cycles with argon. Ethyl vinyl ether was then injected as a catalyst poison to deactivate residual catalyst species. For reactions performed in 75 and 300 mL reactors, ethyl vinyl ether was added via a dropping funnel equipped with pressure equalization; afterwards, the reactor was cooled in an ice bath and purged three times with argon.

Samples were taken at the end of the reactions and, in some cases, during reaction progress. Final samples were collected directly from the reactor vessel, whereas samples taken during the reaction were withdrawn via a sampling valve. For samples collected during reaction progress, ethyl vinyl ether was added immediately after withdrawal to deactivate residual catalyst species. Each sample consisted of 292.5 mg of reaction mixture combined with 25 mg of tetradecane as internal standard and 682.5 mg of diethyl ether as solvent. Gas chromatographic (GC) analyses were performed using an Agilent Intuvo 9000 GC equipped with a flame ionisation detector (FID) and an HP‐5 column (Agilent Technologies). Further details on column specifications and data acquisition are provided in the Supporting Information, together with representative chromatograms.

## 3 Results and Discussion

Since metathesis forms the basis of the value chain illustrated in Figure 2, optimisation of this reaction was carried out first. To enable a systematic evaluation, the complex reaction network shown in Scheme 1 must be described to define the terms main products and by‐products. The reaction network is complex because renewable methyl oleate sources consist of mixtures containing not only methyl oleate but also various polyunsaturated methyl esters such as linoleic acid methyl ester and their derivatives. These can likewise act as substrates for cross‐metathesis with 2‐butene, leading to additional products alongside methyl 9‐undecenoate. As polyunsaturated methyl esters increase the complexity of the reaction network even more, semi‐hydrogenated methyl oleate feedstocks were used [11]. Since hydrogenation does not occur selectively at a single double‐bond position, new potential substrates are formed that may contain a single double bond not only at carbon 9 but also at positions 12 or 15, also contributing to the large number of possible products.

**SCHEME 1:**
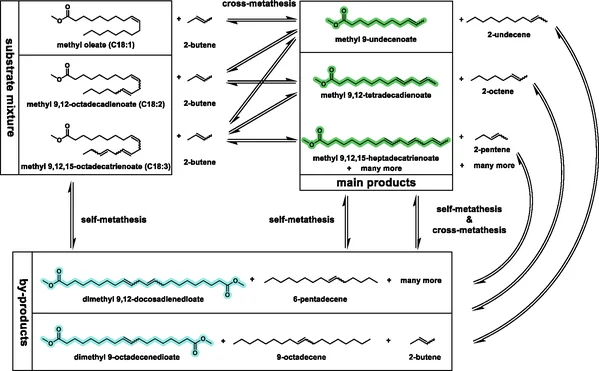
Illustration of the complex reaction network of the cross‐metathesis of local methyl oleate feedstocks with 2‐butene, showing representative examples of main and by‐products.

In addition to the main reaction, the cross‐metathesis between 2‐butene and one of these methyl esters, side reactions such as self‐metathesis or cross‐metathesis between two ester molecules can occur, forming diesters considered by‐products. Furthermore, olefins produced during the metathesis may undergo additional metathesis reactions either with remaining methyl ester substrates or with other olefins, thereby expanding the product portfolio even further. Scheme 1 illustrates the desired main reaction together with several representative undesired side reactions, leading to examples of by‐products.

Not only because of the complex reaction network and the numerous possible side products but also because cross‐metathesis represents the basis of the entire value chain, several parameters were optimised first to maximise the yield of the main product and minimise the formation of by‐products.

### 3.1 Synthesis and Isolation of Methyl 9‐Undecenoate as Platform Chemical

The first step of the value chain focuses on establishing access to methyl 9‐undecenoate as the central platform chemical via the cross‐metathesis of methyl oleate with 2‐butene. Preliminary investigations showed that UltraCat was the most suitable catalyst for the cross‐metathesis reactions conducted in this study and it exhibits high tolerance towards impurities present in the substrate. UltraCat is a ruthenium phenylindenylidene complex featuring two cyclic (alkyl)(amino)carbene (CAAC) ligands. Its structure is provided in Figure S1. The first reaction parameter investigated was temperature as it strongly influences catalyst activity and stability [12, 13, 14]. The optimisation reactions were carried out in a 20 mL pressure autoclave under the following conditions: 11.7 mmol of methyl oleate (soybean oil methyl ester, semi‐hydrogenated, corresponding to the amount of double bonds), 1000 ppm UltraCat, a methyl oleate to 2‐butene ratio of 1:1, reaction time of 3 h, and 500 rpm. All reactions were performed neat, and the compositions of the soybean oil methyl ester and the crude 2‐butene mixture used are provided in Tables S2 and S3, respectively. The influence of temperature was investigated within a range of 20–100 °C to determine its effect on catalytic performance. The results of this temperature screening are shown in Figure S13.

The temperature screening showed that low temperatures limit catalytic activity, whereas excessively high temperatures reduce catalyst performance. At a reaction temperature of 20 °C, the lowest conversion of 24% was obtained. Increasing the temperature to 40 °C, the conversion increased to 82%, while the selectivity remained at 61%. At 60 and 80 °C, similarly high conversions of 85% were achieved with selectivities of 71% and 70%, respectively. A further increase in temperature to 100 °C resulted in partial catalyst deactivation, as both conversion and selectivity slightly decreased. Consequently, 80 °C was selected for subsequent reactions as this temperature provides high catalytic performance while reducing the viscosity of the neat methyl ester mixture [15].

Next, the catalyst loading was investigated; loadings between 250 and 5000 ppm were examined (Figure S14). Increasing the catalyst loading from 1000 to 5000 ppm did not improve conversion or selectivity. The conversion remained constant while the selectivity decreased. When the catalyst loading was reduced, the conversion slightly declined from 85% at 1000 ppm to 78% at 250 ppm, whereas the selectivity increased from 70% at 1000 ppm to 72% at 250 ppm. This behaviour can be explained by the competing reaction pathways illustrated in Scheme 1. A lower concentration of active catalyst species reduces the probability of secondary metathesis reactions, in which the desired main product undergoes further transformation, for example, through self‐metathesis or cross‐metathesis with other reaction products [16]. Due to the higher selectivity and the markedly reduced catalyst concentration, which improves the overall economic efficiency of the process, a catalyst loading of 250 ppm was selected for further investigations.

Finally, the methyl oleate to 2‐butene ratio was adjusted to suppress by‐product formation and increase selectivity [17, 18]. Ratios of methyl oleate to 2‐butene of 1:1, 1:2, and 1:3 were investigated (Figure S15). Increasing the proportion of 2‐butene enhanced the selectivity from 70% at a ratio of 1:1 to 84% at a ratio of 1:3, while the yield of by‐products decreased from 21% to 11%.

Although higher 2‐butene ratios could not be investigated due to instrumental limitations and restricted availability in this study, the 1:3 ratio provided the best compromise between selectivity, reduced by‐product formation, and practical feasibility. Based on these results, 80 °C, 250 ppm UltraCat, and a methyl oleate to 2‐butene ratio of 1:3 were defined as standard conditions for all subsequent experiments.

Using the reaction parameters established in the preliminary investigations, additional local methyl oleate feedstocks were examined to demonstrate the broad applicability of the reaction system to various sources of methyl oleate. In addition to semi‐hydrogenated soybean methyl ester, semi‐hydrogenated methyl esters derived from sunflower and rapeseed oil, as well as semi‐hydrogenated triglycerides, were investigated. Furthermore, methyl 9‐decenoate and pure methyl oleate were also studied (Figure 3). The detailed composition of all substrates is provided in Table S2.

**FIGURE 3:**
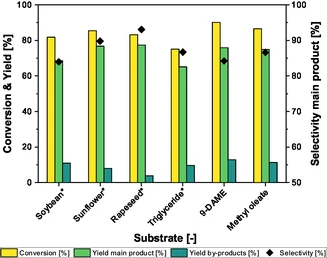
Comparison of various local methyl oleate feedstock in the cross‐metathesis with 2‐butene (* = semi‐hydrogenated) (composition of all substrates is provided in Table S2). Conditions: 11.7 mmol of methyl oleate feedstock, 250 ppm UltraCat, methyl oleate to 2‐butene ratio of 1:3, *T* = 80 °C, *t* = 3 h, 500 rpm. All yields and conversions were determined via GC‐FID with the internal standard *n*‐tetradecane.

As shown in Figure 3, comparable or even improved results relative to soybean methyl ester are obtained when varying the substrate. Conversions of up to 90% were achieved with 9‐DAME. Among the semi‐hydrogenated methyl oleate feedstocks investigated, sunflower as feedstock exhibited the highest conversion of 85%. The highest selectivity was observed for rapeseed methyl ester, reaching 93%, with a main product yield of 77%. The second‐highest selectivity was obtained with semi‐hydrogenated sunflower methyl ester of 90 % at a comparable main product yield of 77%. The observed differences in conversion and selectivity may be attributed to variations in the composition of the respective feedstocks. Differences in the amount of polyunsaturated components affect the catalyst reactivity and selectivity for cross‐metathesis [11, 19]. Furthermore, differences in the content of residual impurities associated with the different plant oil feedstocks may influence catalyst performance. Nevertheless, these results demonstrate the robustness of the reaction system and highlight its potential to be extended to additional locally available methyl oleate feedstocks.

For further investigations, sunflower methyl ester was selected as the most suitable feedstock for producing methyl 9‐undecenoate in sufficient quantity and purity for downstream functionalisation. Although rapeseed methyl ester provided slightly higher selectivity, a broader product distribution was observed, resulting in lower formation of the desired platform molecule, methyl 9‐undecenoate.

To obtain sufficient material for isolation and subsequent transformations, the cross‐metathesis was scaled up using sunflower methyl ester as feedstock. The reaction initially carried out in a 20 mL pressure autoclave was scaled up first to a 75 mL autoclave (six‐fold increase relative to the 20 mL setup) and subsequently to a 300 mL reactor (twentyfold increase relative to the 20 mL setup).

During the scale‐up, no significant changes in conversion(±5.9%), yield (±4.2%), or selectivity (±1.5%) were observed. This demonstrates a smooth and reproducible scale‐up of the reaction to larger batch sizes. Furthermore, sufficient reaction mixture was obtained to isolate the desired product, methyl 9‐undecenoate, from the crude mixture by distillation using a rectification column, achieving a purity of up to 99.4%. The obtained methyl 9‐undecenoate can subsequently be converted, as shown in Figure 2, through isomerising functionalisation reactions and further reactions into symmetric or non‐symmetric polymer precursors.

### 3.2 Isomerising Functionalisation of Methyl 9‐Undecenoate Towards Linear C_12_ Intermediates

Having established and isolated methyl 9‐undecenoate as the central renewable platform chemical, its suitability for accessing C_12_ polymer precursors was evaluated through subsequent isomerising functionalisation reactions and further functionalisation reactions. The first route focused on symmetric C_12_ polymer precursors, using isomerising methoxycarbonylation as the key isomerising functionalisation step to convert methyl 9‐undecenoate into the corresponding linear C_12_ dimethyl ester.

### 3.3 Symmetrical Functionalisation

#### 3.3.1 Isomerising Methoxycarbonylation

As shown in Figures 2 and 4, symmetric polymer precursors were obtained through isomerising methoxycarbonylation. The catalyst system employed, consisting of the catalyst precursor Pd_2_(dba)_3_ and the ligand 1,2‐DTBPMB, as well as the reaction conditions, was used with methanesulphonic acid as an acid additive. The reaction was carried out using methanol as both the solvent and reactant at 95 °C under a CO pressure of 30 bar for 23 h. These conditions were adapted from literature procedures [20, 21, 22, 23, 28, 29]. The methyl 9‐undecenoate used had a purity of 95.7%. Other components present included methyl 9‐decenoate (4%), and short‐chain alkenes (0.3%). The yields of the linear products obtained from both methyl 9‐undecenoate and methyl 9‐decenoate are reported collectively, as are the yields of the corresponding branched products of these two methyl esters.

**FIGURE 4:**
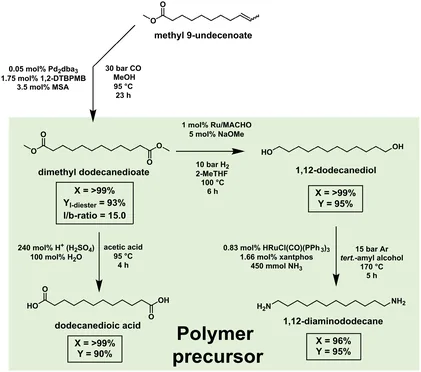
Overview of symmetric C_12_ polymer precursors derived from the renewable platform chemical methyl 9‐undecenoate via isomerising methoxycarbonylation and subsequent functionalisation reactions. Reaction conditions, conversions and yields are provided for each transformation [20, 21, 22, 23, 24, 25, 26, 27, 28, 29].

After 23 h, >99% conversion was achieved, yielding 93% of the linear methoxycarbonylation product and 6% of branched products with a linear (l)‐to‐branched (b) ratio (l/b‐ratio) of 15. The detailed reaction profile is shown in Figure S17, together with the reaction conditions.

The results obtained are comparable to those reported in literature; therefore, methyl 9‐undecenoate can be considered an equivalent substrate to methyl 10‐undecenoate and methyl oleate [21, 22]. From the synthesized C_12_‐dimethyl ester, 1,12‐dimethyl dodecanedioate, three further polymer precursors can be derived through functionalisation reactions, as shown in Figures 2 and 4.

#### 3.3.2 Ester Reduction

To expand the portfolio of polymer precursors, ester reduction of 1,12‐dimethyl dodecanedioate to 1,12‐dodecandiol was investigated using reaction conditions adapted from literature [24, 26]. The reaction was performed using the catalyst Ru‐MACHO in combination with NaOMe as a base and 2‐Me‐THF as the solvent. The reaction was carried out at 100 °C under a H_2_ pressure of 10 bar for 6 h at 500 rpm. Complete conversion (>99%) was achieved, with a yield of 95% for the diol. The resulting 1,12‐dodecandiol can serve not only as a polymer precursor in itself but also as a substrate for further functionalisation reactions.

#### 3.3.3 Alcohol Amination

A subsequent product derived from 1,12‐dodecandiol is 1,12‐dodecandiamine, which can be synthesised via alcohol amination as shown in Figures 2 and 4 using literature procedure [27, 30]. The reaction was performed using RuHCl(CO)(PPh_3_)_3_ as the catalyst precursor and Xantphos as the ligand, with *tert*‐amyl alcohol as the solvent. The reaction was carried out at 170 °C under an initial argon pressure of 15 bar using 30 equivalents of NH_3_ and a stirring rate of 900 rpm. After a reaction time of 5 h, a conversion of 96% was achieved with a yield of 95% for 1,12‐dodecandiamine, values comparable to those reported in literature [27]. The conversion‐time profile is depicted in Figure S18.

#### 3.3.4 Ester Hydrolysis

To further expand the range of symmetric C_12_ polymer precursors, ester hydrolysis of 1,12‐dimethyl dodecanedioate to form 1,12‐dodecanedioic acid was investigated. As no suitable reaction conditions reported in the literature were applicable to the investigated reaction system, new parameters were developed to enable efficient hydrolysis using sulphuric acid as the catalyst, acetic acid as the solvent, and water as a reactant. The reaction was carried out at 95 °C for 4 h. Complete conversion (>99%) was achieved with a yield of 90% for 1,12‐dodecanedioic acid, and minor amounts (2%) of mono‐hydrolysed substrate and side products (9%) were also formed.

### 3.4 Non‐Symmetrical Functionalisation

#### 3.4.1 Isomerising Hydroformylation

As illustrated in Figures 2 and 5, based on the platform molecule methyl 9‐undecenoate, not only symmetric but also non‐symmetric polymer precursors can be generated via an initial isomerising hydroformylation step forming methyl 12‐oxododecanoate, followed by subsequent functionalisation reactions. An auto‐tandem catalytic system comprising Rh/BiPhePhos was employed, based on robust optimised conditions previously established for the isomerising hydroformylation of internal olefins [33, 34, 35]. Using DoE‐optimised reaction parameters (Set 1) in toluene at 118 °C and a CO/H_2_ pressure of 12 bar, a conversion of 74% of methyl 9‐undecenoate was achieved after 3 h, with a yield of 65% for the desired linear product, methyl 12‐oxododecanoate, with an l/b‐ratio of 21. Applying modified reaction parameters (Set 2), designed to further increase selectivity towards the linear aldehyde, in toluene at 105 °C and a CO/H_2_ pressure of 5 bar resulted in a conversion of 78% after 22 h, with a yield of 71% of the desired linear aldehyde, methyl 12‐oxododecanoate, and an l/b‐ratio of 30. Detailed conversion‐time profiles are presented in Figures S19 and S20.

**FIGURE 5:**
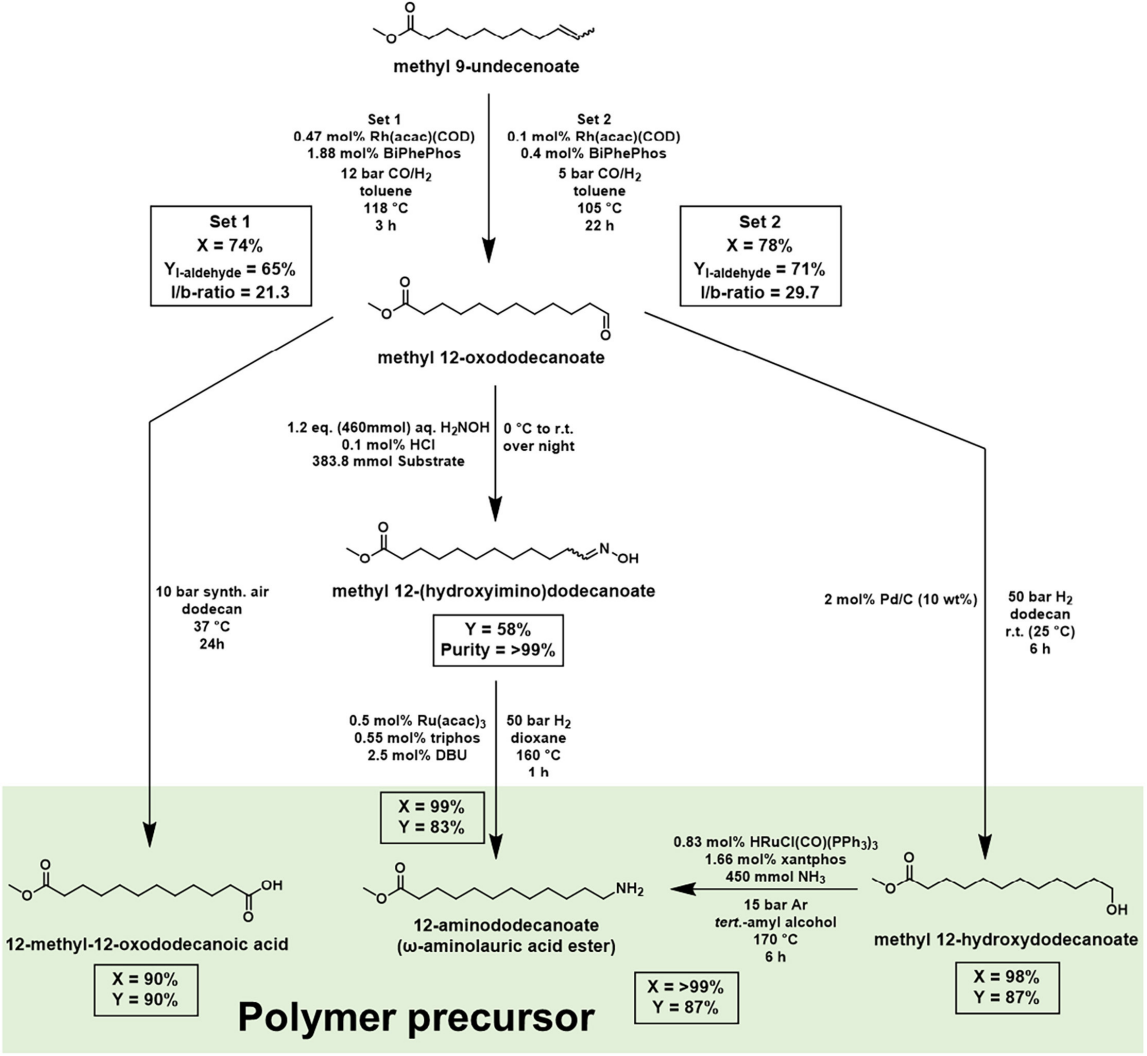
Overview of non‐symmetric C_12_ polymer precursors derived from the renewable platform chemical methyl 9‐undecenoate via isomerising hydroformylation and subsequent functionalisation reactions. Reaction conditions, conversions and yields are provided for each transformation [27, 31, 32, 33].

#### 3.4.2 Oxidation

Starting from methyl 12‐oxododecanoate, 12‐methoxy 12‐oxododecanoic acid was formed via oxidation using adapted reaction conditions from literature [31, 32]. The reaction was carried out in toluene at 37 °C under a pressure of 10 bar synthetic air for 24 h. Under these conditions, a conversion of 90% and a yield of 90% of 12‐methoxy 12‐oxododecanoic acid were achieved.

#### 3.4.3 Hydrogenation

Another functionalisation route proceeds via hydrogenation, yielding methyl 12‐hydroxydodecanoate, which serves both as polymer precursor itself and as starting material for further transformations. The reaction was performed in toluene using Pd/C (10 wt%) as the catalyst under a H_2_ pressure of 50 bar at room temperature. After 6 h of reaction time, a conversion of 98% was achieved, with a yield of 87% of methyl 12‐hydroxydodecanoate.

#### 3.4.4 Alcohol Amination

To introduce amino functionalities, methyl 12‐hydroxydodecanoate was converted via alcohol amination into methyl 12‐aminododecanoate or ω‐aminolauric acid ester, following literature procedures [27, 30]. The reaction was performed using RuHCl(CO)(PPh_3_)_3_ as the catalyst precursor and Xantphos as the ligand, with *tert*‐amyl alcohol as the solvent. The reaction was carried out at 170 °C under an initial argon pressure of 15 bar, using 30 equivalents of NH_3_ and 900 rpm stirring rate. In addition to the desired methyl 12‐aminododecanoate, the corresponding amino acid, 12‐aminododecanoic acid, was also formed. The combined yield of the two aminated products was 87% at a conversion of >99% after 2 h. The corresponding reaction profile is shown in the Figure S21.

#### 3.4.5 Oxime Formation and Hydrogenation

An alternative route towards polymer precursors containing amino functionalities is the oxime formation through the condensation of hydroxylamine and methyl 12‐oxododecanoate in the presence of catalytic amounts of hydrochloric acid, followed by hydrogenation of the resulting oxime using a Ru(acac)_3_/triphos catalyst system in the presence of DBU, according to the literature [9]. The oxime intermediate was obtained with a purity of 99.2%. The hydrogenation was performed in dioxane at 160 °C under 50 bar H_2_, resulting in a conversion of 99% and an overall yield of 83% of methyl 12‐aminododecanoate and amine intermediates convertible to methyl 12‐aminododecanoate (Figure S22).

### 3.5 Comparison of the Synthetic Routes to C_12_ Polymer Precursors

To assess the relative performance of the different synthetic routes towards C_12_ polymer precursors, the overall yields (ΣY%) and atom economies (AE%) were calculated starting from the new platform molecule, methyl 9‐undecenoate. The corresponding values for all investigated routes are provided in Figure S23. As the further functionalisation reactions were primarily intended to demonstrate the feasibility of the proposed value chain, the routes were not comprehensively optimised, and their ranking does not necessarily reflect their maximum achievable performance.

Among the symmetric C_12_ precursors, the route towards 1,12‐dimethyl dodecanedioate exhibited the highest overall yield and atom economy, both reaching 93%. The routes towards 1,12‐dodecanediol and 1,12‐dodecanediamine afforded overall yields of 88% and 84%, with atom economies of 68% and 57%, respectively. The route towards 1,12‐dodecanedioic acid achieved an overall yield of 84% and an atom economy of 70%.

The non‐symmetric C_12_ precursors generally exhibited lower overall yields and atom economies, primarily owing to the lower yield of the initial isomerising hydroformylation step. The reaction time was 3 h for Set 1 and 22 h for Set 2. Despite the slightly higher yield of the linear aldehyde obtained with Set 2 and resulting higher overall yields, the shorter reaction time makes Set 1 more likely to be attractive for further optimisations. Oxidation afforded the highest overall yields among the non‐symmetric precursors, at 59% and 64% for Sets 1 and 2, respectively, whereas hydrogenation to methyl 12‐hydroxydodecanoate provided the highest atom economies, at 57% and 62%, respectively. For methyl 12‐aminododecanoate, the route via aldehyde hydrogenation followed by alcohol amination was more favourable, achieving overall yields of 49% and 54% and atom economies of 46% and 50% for Sets 1 and 2, respectively. The alternative oxime route gave overall yields of 31% and 34% and atom economies of 27% and 30%, respectively, but may offer an advantage through easier purification of the oxime intermediate.

Catalyst recovery and recycling could further improve process efficiency, particularly in the initial isomerising functionalisation steps. In isomerising methoxycarbonylation, selective product crystallisation has been demonstrated for the simultaneous separation of the linear diester and recovery of the homogeneous catalyst in the conversion of methyl oleate [23, 36]. Biphasic systems have also been investigated for catalyst recycling in palladium‐catalysed methoxycarbonylation of terminal alkenes [37]. For isomerising hydroformylation, studies using homogeneous Rh/BiPhePhos catalysts have addressed catalyst recycling using thermomorphic solvent systems and distillation [38, 39]. Although the latter studies focused on internal olefins rather than fatty acid methyl esters, they provide relevant starting points for evaluating catalyst recovery and reuse in the present system.

Further optimisation of the downstream functionalisation reactions and reaction conditions, together with improved product recovery and catalyst recycling, will be required to enhance the overall process efficiency of the different routes. The present comparison provides a basis for identifying promising pathways, while further research is needed to realise their full potential.

## 4 Conclusion

In this study, we have established methyl 9‐undecenoate as a renewable platform chemical accessible from locally available methyl oleate feedstocks via cross‐metathesis with 2‐butene. This intermediate represents a key building block for the synthesis of a broad portfolio of C_12_ polymer precursors derived from renewable resources. Optimisation of the metathesis reaction established robust conditions that enable high conversions of 82% and selectivities of 84% to the main product while maintaining scalability. The successful scale‐up of the reaction from 20 to 300 mL reactors confirmed the technical feasibility of the process and allowed the isolation of methyl 9‐undecenoate in high purity of more than 99% for subsequent transformations.

Building on the platform molecule methyl 9‐undecenoate, the downstream isomerising functionalisation reactions methoxycarbonylation and hydroformylation were successfully carried out, providing access to the versatile intermediates 1,12‐dimethyl dodecanedioate and methyl 12‐oxododecanoate. Isomerising methoxycarbonylation achieved >99% conversion with a linear diester yield of 93%, and an l/b‐ratio of 15, whereas isomerising hydroformylation achieved conversions of up to 78% with a linear aldehyde yield of 71% and an l/b‐ratio of 29.7. Both intermediates serve as central platform chemicals for further transformations. From these intermediates, a diverse range of symmetric and non‐symmetric polymer precursors were synthesised, including diols, diamines, dicarboxylic acids, as well as amino‐, alcohol‐, and carboxylic acid‐functionalizedd esters. The results demonstrate the versatility of the developed reaction sequence and its suitability for generating a broad spectrum of C_12_‐based building blocks relevant for polymer chemistry.

The platform molecule methyl 9‐undecenoate produced in this work exhibits a renewable carbon index (RCI) of >0.8. The derived polymer precursors obtained along this pathway retain high renewable carbon contents, reaching RCI values of up to 0.76. These results highlight the potential of the developed approach to contribute to more sustainable production routes for polymer precursors.

Overall, the study establishes a complete and scalable value chain from local plant oil feedstocks via methyl 9‐undecenoate as the central renewable platform chemical to a diverse portfolio of renewable C_12_ polymer precursors. By combining cross‐metathesis with subsequent isomerising functionalisation reactions, the presented approach provides a practical route towards sustainable platform chemicals that may serve as building blocks for future bio‐based polymer materials.

## Funding

This work was supported by the Fachagentur Nachwachsende Rohstoffe (2219NR355).

## Conflicts of Interest

The authors declare no conflict of interest.

## Supporting information

Detailed information is given on the chemicals used, analysis and experimental procedures. Further experimental results are also given.

## Data Availability

The data that support the findings of this study are available from the corresponding author upon reasonable request.
