# Supplementary material for: Establishing Methyl 9‐Undecenoate as a Renewable Platform Molecule From Local Plant Oils for Catalytic Access to C12‐Polymer Precursors
Source: ChemSusChem. 2026 Nov 14;19(22):e71234. doi: 10.1002/cssc.71234 (PMC13769245; doi:10.1002/cssc.71234)
Supplement: Supplementary file 1 [file CSSC-19-e71234-s001.pdf]

Supporting Information

Renewable Methyl 9-Undecenoate as a Platform Chemical for the Development of a Sustainable Value Chain from Local Plant Oils to C_{12} Polymer Precursors

Jerome Hommes,[1] Jan H. Behler,[1] Hendrik S. Lapainis,[1] Dieter Vogt[1] and Thomas Seidensticker[1,*]

[1]TU Dortmund University, Department for Biochemical and Chemical Engineering, Laboratory of Industrial Chemistry, Emil-Figge-Straße 66, 44227 Dortmund, Germany

*Dr. Thomas Seidensticker, email: thomas.seidensticker@tu-dortmund.de, phone: +49 231 755 2310

Table of Contents

Supporting Information ... 1

Table of Contents ... 1

1. General Methods and Reagents ... 3

1.1. Structure of the Metathesis Catalyst UltraCat ... 6

1.2. Composition of Methyl Oleate Feedstocks ... 6

1.3. Composition of 2-Butene Mixture ... 6

1.4. General Procedures ... 7

Isomerising Methoxycarbonylation ... 7

Ester-Reduction ... 7

Alcohol Amination ... 8

Ester Hydrolysis ... 9

Isomerising Hydroformylation ... 9

Oxidation ... 10

Hydrogenation ... 10

Oxime Formation & Hydrogenation ... 11

2. Analytics 12
2.1. Specifications of the column 12
2.2. Heating Profiles Used 12
2.3. Representative Chromatogram 14
3. Additional Results 20
3.1. Cross-Metathesis Optimisation 20
3.2. Isomerising Methoxycarbonylation 22
3.3. Alcohol Amination of 1,12-dodecandiol 23
3.4. Isomerising Hydroformylation 23
3.5. Alcohol Amination of methyl 12-hydroxydodecanoate 24
3.6. Oxime Hydrogenation 25
4. References 26

1. General Methods and Reagents

All chemicals used for the experiments were stored under argon, and the liquid chemicals were degassed with argon dispersed by a frit for at least 30 min in an ultrasonic bath or by applying vacuum for at least 30 min in an ultrasonic bath.

Table S1: Chemicals used in this work.

Chemical	Manufacturer	Purity [w%]
1,4-Dioxane	Merck	99
1,12-Diamino dodecane	Merck	98
1,12-Dimethyl dodecandioate	self made	>99
1,12-Dodecandiol	Thermo scientific	99
1,12-Dodecandioic acid	Thermo scientific	99
1,2-DTBPMB 1,2-Bis(di-*tert*-butylphosphinomethyl)benzene	Merck	≥99
2-Butene	Evonik Oxeno GmbH& Co.KG	-
2-Methyl 2-butanol	abcr GmbH + Co. KG	98
2-Me-THF 2-Methyltetrahydrofuran	Carl Roth GmbH + Co. KG	99
12-hydroxydodecanoate	self made	73.3
Acetic acid	Carl Roth GmbH + Co. KG	>99
Acetylacetonato(1,5-cyclooctadiene)rhodium(I)	Merck	99
Aluminium oxide	Carl Roth GmbH + Co. KG	>99
Ammonia	Messer SE	>99
tert-amyl alcohol	TCI	98
Argon	Messer SE	99.996
BiPhePhos/Oxophos17 6,6′-[(3,3′-Di-*tert*-butyl-5,5′-dimethoxy-[1,1′-biphenyl]-2,2′-diyl)bis(oxy)]bis(6*H*-dibenzo[*d,f*][1,3,2]dioxaphosphepine)	Evonik Oxeno GmbH& Co.KG	-
Carbon monoxide	Messer SE	99.99
Carbonylchlorohydridotris (triphenylphosphine)ruthenium(II)	Thermo scientific	>99
n-Decane	Merck	99

Deionised water	-	>99
DBU<br>Diazabicycloundecen	Carl Roth GmbH + Co. KG	98
Diethyl ether	Carl Roth GmbH + Co. KG	99.5
n-Dodecane	Thermo scientific	99
Ethoxyethane	Thermo scientific	99.5
Ethyl vinyl ether	TCI	>98
Hydrochloric acid	Thermo scientific	37
Hydrogen	Messer SE	99.999
Hydroxylamine	Fluorochem	50
Isopropanol	VWR	>99
Methanesulphonic acid	Carl Roth GmbH + Co. KG	99.5
Methanol	Carl Roth GmbH + Co. KG	99.9
Methyl 9-decenoate	Verbio SE	>95
Methyl 9-undecenoate	self made	95.5
Methyl oleate	Merck	99
N_2	Messer SE	99.5
Oxime<br>Methyl 12-(hydorxyimino)dodecanoate	self made	99.2
Pd/C (10 wt%)	Thermo scientific	>99.9
n-Pentane	Carl Roth GmbH + Co. KG	99
Rh(acac)(COD)<br>Acetylacetonato(dicarbonyl)rhodium(I)	Merck	99
$RuHCl(CO)(PPh_3)_3$<br>Carbonylchlorhydridotris<br>(triphenylphosphin)ruthenium(II)	Merck	99.9
$Ru(acac)_3$<br>Ruthenium(III) acetylacetonate	Merck	97
Ru-MACHO<br>Carbonylchlorohydrido{bis[2-<br>(diphenylphosphinomethyl)ethyl]amino}<br>ethyl]amino}ruthenium(II)	abcr GmbH + Co. KG	98
Sodium methanolate	Merck	98
Sulforic acid	Thermo scientific	38
Syngas (1:1)	Messer SE	50 ± 2 mol% H_2,<br>50 ± 2 mol% CO,

		H_2 99.999 , CO 99.997
Synthetic air	Messer SE	99.999%<br>20.5 Vol% O_2,<br>79.5 Vol% N_2
n-Tetradecane	Merck	99
Toluene	Thermo scientific	99
Triphos<br>1,3-Bis(diphenylphosphino)-2-(diphenylphosphino)methyl-2-methylpropane, Triphos	BLDpharm	97
$Pd_2(dba)_3$<br>Tris(dibenzylideneacetone)dipalladium	Thermo scientific	97
UltraCat<br>Bis(1-(2,6-diethylPh)-3,5,5-trimethyl-3-phenylpyrrolidin-2-ylidene)(3-Ph-1H-inden-1-ylidene)ruthenium(II) dichloride	BLD PHARMATECH GmbH	≥95
Xantphos<br>(9,9-Dimethyl-9*H*-xanthene-4,5-diyl)bis(diphenylphosphane)	carbolution chemicals	98

1.1. Structure of the Metathesis Catalyst UltraCat

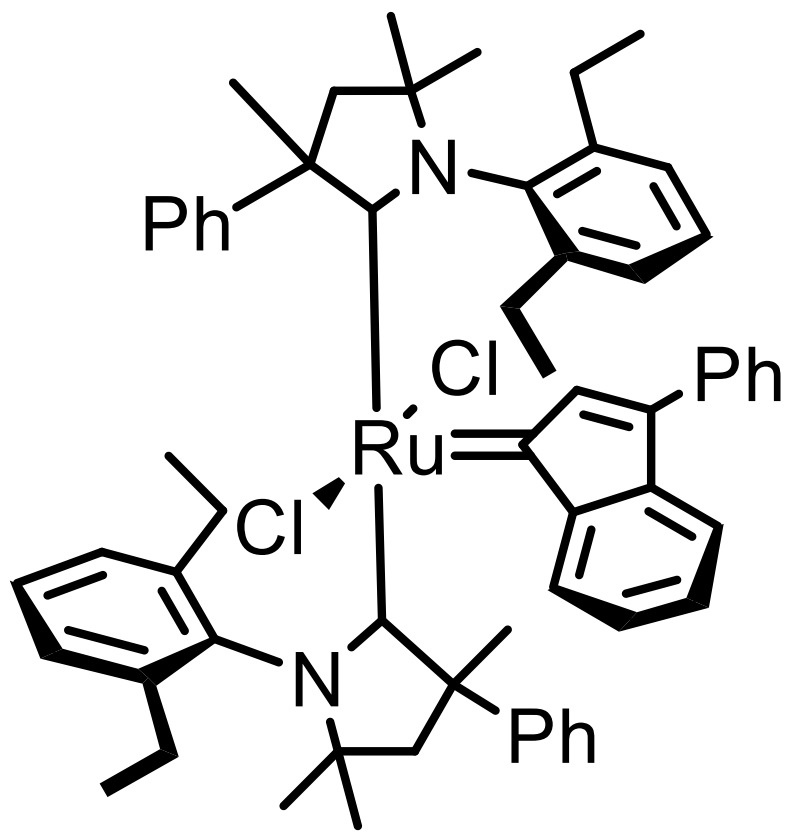

Figure S1: Structure of the metathesis catalyst UltraCat (Bis(1-(2,6-diethylPh)-3,5,5-trimethyl-3-phenylpyrrolidin-2-ylidene)(3-Ph-1H-inden-1-ylidene)ruthenium(II) dichloride) used for the cross-metathesis of methyl oleate with 2-butene.[1]

1.2. Composition of Methyl Oleate Feedstocks

Table S2: Composition of the used methyl oleate feedstocks.

Methyl oleate feedstock	C16:0 [%]	C18:0 [%]	C18:1 [%]	C18:2 [%]	C18:3 [%]
Soybean	11.2	0.0	79.0	2.9	6.9
Sunflower	3.7	4.2	91.9	0.2	0.0
Rapeseed	5.0	11.0	81.4	1.9	0.7
Triglyceride	4.6	14.3	80.6	0.5	0.0
Methyl oleate	0	0	>99	0	0

1.3. Composition of 2-Butene Mixture

Table S3: Composition of the industrial available 2-butene mixture.

Component	Amount [%]
n-Butane	62.4
trans-2-Butene	23.7
cis-2-Butene	10.7
1-Butene	2.1
Isobutane	0.6
Neopentane	0.5
Isopentane	0.1

1.4. General Procedures

Isomerising Methoxycarbonylation

Prior to the experiment, the methanol used was dried over sodium sulphate overnight. Both methyl 9-undecenoate and methanol were degassed under argon in an ultrasonic bath for at least 30 min before use. All glassware used was heated under vacuum and subsequently purged with argon, this procedure was repeated three times. Inside the glovebox, 1,2-DTBPMB (116.7 mg, 0.295 mmol) together with $Pd_2(dba)_3$ (7.7 mg, 0.008 mmol) was weighed into a Schlenk flask, and subsequently methanol (20 mL) was added under a counterflow of argon. The catalyst mixture was stirred overnight at room temperature (20 °C). Afterwards, methanesulfonic acid (39 µL, 0.59 mmol) was added to the catalyst mixture under an argon counterflow. After brief stirring (10 min), the catalyst solution was transferred under argon counterflow into the reactor.

Methyl 9-undecenoate (16.9 mmol) and methanol (10 mL) were then added under an argon counterflow into the dropping funnel with pressure equalisation. A CO pressure of 20 bar was applied, and the reactor was heated while stirring (1000 rpm) to 95 °C. Once the reaction temperature had been reached, the reaction was initiated by swiftly opening the dropping funnel to transfer the substrate into the reactor, subsequently, the CO pressure was increased to 30 bar.

To monitor reaction progress, samples were periodically withdrawn from the reactor *via* a sampling valve and analysed by gas chromatography (GC). A typical GC sample for methoxycarbonylation consisted of 475 mg reaction mixture, 25 mg *n*-dodecane as internal standard, and 500 mg isopropanol as solvent. Gas chromatographic analyses were performed on an Agilent 7890A GC equipped with a flame ionisation detector (FID) and an HP-5 column (Agilent Technologies). Further details regarding the column specifications and data acquisition are provided in Table S4 and Table S5.

After a reaction time of 23 h, the reactor was placed in an ice bath. Thereafter, the gas phase was vented and flushed three times with nitrogen (10 bar). The reactor was then opened, and the reaction solution collected for storage. The reactor was thoroughly cleaned after each reaction.

Ester-Reduction

The 2-Me-THF used was degassed with argon in a ultrasonic bath for at least 30 min prior to the experiment. Inside a glovebox, the base NaOMe (0.029 mmol, 3.3 mg) was weighed into a 20 mL pressure autoclave. In a Schlenk flask, heated under vacuum and purged with argon

(three times), the substrate (0.57 mmol, 148 mg) and Ru-MACHO (0.0055 mmol, 3.4 mg) were dissolved in 3 mL 2-Me-THF and subsequently transferred under argon counterflow into the reactor. Hydrogen pressure was then increased to 10 bar, and the reactor was placed in a preheated heating block (100 °C) and stirred at 500 rpm, marking the start of the reaction.

After a reaction time of 6 h, the reactor was placed in an ice bath, pressure was carefully released and flushed three times with nitrogen. The reactor was then opened, a GC sample withdrawn, the storage solution collected, and the reactor cleaned thoroughly.

A typical GC sample consisted of 450 mg reaction mixture, 25 mg *n*-dodecane as internal standard, and 500 mg 2-Me-THF as solvent. GC analyses were performed on an Agilent 7890A GC equipped with a FID and an HP-5 column (Agilent Technologies). Further details regarding column specifications and data acquisition are provided in Table S4 and Table S5.

Alcohol Amination

$RuHCl(CO)(PPh_3)_3$ (121.3 mg, 0.125 mmol) and Xantphos (147 mg, 0.25 mmol) were added to a 75 mL pressure reactor. After sealing and performing three argon/vacuum cycles, 1,12-dimethyl dodecanedioate (1533 mg, 7.5 mmol) or methyl 12-hydroxydodecanoate (4716 mg, 15 mmol) and *tert*-amyl alcohol (37.5 mL) were added into the dropping funnel with with pressure equalisation under an argon counterflow. Subsequently, an argon pressure of 15 bar was applied to the reactor and ammonia (12.63 mL, corresponding to 30 equivalents) was added to the reactor using a CETONI Nemesys M syringe pump.

After heating the catalyst mixture to 170 °C and stirring it at 900 rpm for 20 min at 170 °C, the substrate was introduced into the reactor by quickly opening the dropping funnel. GC-samples were withdrawn periodically via a sampling valve. Typical GC-samples consist of 450 mg of reaction mixture, which was first degassed befor the GC-FID analysis, 25 mg of *n*-dodecane as internal standard and 500 mL of *tert*-amyl alcohol as solvent. GC analyses were performed on an Agilent 7890A GC equipped with a FID and an HP-5 column (Agilent Technologies). Further details regarding column specifications and data acquisition are provided in Table S4 and Table S5.

After a reaction time of 5 h, the reactor was cooled to room temperature in an ice bath and the pressure was carefully released and the reactor was flushed three times with nitrogen (10 bar).

Ester Hydrolysis

1,12-Dimethyl dodecandioate (258.4 mg, 1 mmol), acetic acid (15 mL), deionised water (2 mL) and sulphuric acid (117.7 mg, 1.2 mmol) were added to a Schlenk flask. A short-path distillation was mounted on the Schlenk flask, and the reaction mixture was placed on a pre-heated heating plate (95 °C) and stirred at 600 rpm. During the reaction, a gentle argon flow was applied. GC-samples were withdrawn periodically using a long needle. A typical GC-samples consistes of 450 mg of reaction mixture, the corresponding amount of reaction mixture was taken, and the solvent acetic acid was subsequently removed using a rotary evaporator. The residue was then redissolved in isopropanol until a total mass of 450 mg was reached. In addition, 25 mg *n*-dodecane as internal standard and 500 mg isopropanol as solvent were added. GC analyses were performed on an Agilent 7890A GC equipped with a FID and an HP-5 column (Agilent Technologies). Further details regarding column specifications and data acquisition are provided in Table S4 and Table S5.

Isomerising Hydroformylation

All isomerising hydroformylation experiments were performed in a 75 mL autoclave equipped with a cross-shaped magnetic stir bar and a dropping funnel with pressure equalisation. Prior to each run, the solvent was degassed under argon and sonicated for 30 min. Two Schlenk flasks (flask 1 and flask 2) were evacuated, and purged with argon, this process was repeated three times.

The precursor Rh(acac)(COD) (30.2 mg, 0.097 mmol / 6.45 mg, 0.021 mmol) was added to flask 1 using standard Schlenk techniques, followed by addition of 10 mL of toluene with argon counterflow. In flask 2, BiPhePhos (306 mg, 0.39 mmol / 65.5 mg, 0.083 mmol) was weighed in and dissolved in 30 mL of toluene under identical conditions. The solids in both flasks were dissolved by ultrasonic treatment until completely dissolved (for at least 10 min). Afterwards, the precursor solution from flask 1 was transferred to the ligand solution contained in flask 2 under an argon counterflow. The combined mixture was again sonicated for 10 min to ensure full homogenisation.

Before adding the catalyst mixture to the reactor, it was flushed three times with argon. The prepared precursor/ligand solution was then charged into the reactor under argon counterflow while stirring commenced immediately thereafter. To maintain inert conditions, three pressurisation-depressurisation cycles (PDP) were conducted using nitrogen at 10 bar. Once the dropping funnel had been isolated from the main vessel, three additional PDP cycles were carried out with synthesis gas at 10 bar. Subsequently, half of the intended reaction pressure was applied using synthesis gas and heating initiated, upon reaching the desired temperature,

full reaction pressure was established and catalyst preformation allowed to proceed for one hour to ensure some complex formation.

The substrate, methyl 9-undecenoate (2.4 g, 20.8 mmol), was subsequently added into the dropping funnel. After adjusting the pressure in the dropping funnel to match the reactor pressure *via* its equilibration valve, the reaction was started by quickly adding the substrate through opening the dropping funnel valve towards the reactor.

Throughout each reaction, samples were periodically withdrawn *via* a sampling valve and analysed by GC to monitor the reaction. Each GC-sample consisted of 450 mg reaction mixture, 25 mg *n*-decane serving as internal standard and 500 mg diethyl ether as solvent. GC analyses were performed on an Agilent Intuvo 9000 system equipped with a FID and HP-5 column (Agilent Technologies). Detailed information regarding column specifications and chromatographic parameters is provided in Table S4 and Table S6.

Oxidation

A substrate mixture (2 g), consisting of a reaction solution containing 12 wt% methyl 12-oxododecanoate in dodecane, was added to a 20 mL pressure reactor equipped with a cross-shaped magnetic stir bar. The reactor was then sealed and pressurised with 10 bar synthetic air. The oxidation reaction was considered to start when the reactor was placed in a pre-heated heating block at 37 °C and stirred at 500 rpm. After a reaction time of 24 h, the process was stopped and the system allowed to cool to ambient temperature. Subsequently, the pressure was carefully released, and a GC-sample was prepared.

Each GC-sample consisted of 475 mg of reaction mixture, 25 mg *n*-decane as internal standard, and 500 mg isopropanol as solvent. Gas chromatographic analyses were performed on an Agilent 7890A GC equipped with a FID and an HP-5 column (Agilent Technologies). Further details regarding column specifications and data acquisition are provided in Table S4 and Table S5.

Hydrogenation

The hydrogenation of methyl 12-oxododecanoate was carried out in a 20 mL pressure autoclave equipped with a cross-shaped magnetic stir bar. Prior to use, the reactor was purged with argon by applying three vacuum-argon cycles.

Pd/C (10 wt%) (0.05 mmol Pd, 55 mg) was first introduced into the argon filled reactor. Subsequently, the substrate methyl 12-oxododecanoate (2.58 mmol), dissolved in dodecane

(11.8 wt%, 5 g), was added. The reactor was then pressurised with 50 bar of H_2. The reaction was initiated by placing the reactor in a heating block at room temperature and stirring at 500 rpm.

After a reaction time of 6 h, the reactor was placed in an ice bath. Once cooled, the hydrogen pressure was carefully released and the reactor was purged three times with nitrogen. The reactor was then opened and a GC sample was prepared.

Each GC sample consisted of 475 mg of reaction mixture, 25 mg of *n*-decane as internal standard, and 500 mg of isopropanol as solvent. Gas chromatographic analyses were performed using an Agilent 7890A GC equipped with a flame ionisation detector (FID) and an HP-5 column (Agilent Technologies). Further details regarding column specifications and data acquisition are provided in Table S4 and Table S5.

Oxime Formation & Hydrogenation

Oxime formation

For oxime formation, 145 g (383.8 mmol) of a hydroformylation reaction mixture consisting of a butanol/water mixture containing 60.4 wt% aldehydes was added to a 500 mL round-bottom flask equipped with a magnetic stir bar. The flask was placed in an ice bath and stirred. Hydroxylamine (50% mixture in water) (30.4 g, 460.6 mmol) and HCl (38.9 mg, 0.38 mmol) were slowly added dropwise under stirring. The reaction mixture was stirred overnight while gradually warming to room temperature. After completion of the reaction, the resulting colourless solid was washed three times with pentane and subsequently dried under high vacuum. The obtained oxime was isolated with a purity of 99.2%.

Oxime hydrogenation

The hydrogenation of the oxime was carried out in a 75 mL autoclave equipped with a cross-shaped magnetic stir bar and a dropping funnel with pressure equalisation. The reactor was sealed and evacuated. In a flame-dried Schlenk flask under argon counterflow, $Ru(acac)_3$ (0.038 mmol, 14.9 mg) and triphos (0.041 mmol, 26.6 mg) were weighed in and dissolved in 19.5 mL of dioxane. Diazabicycloundecen (DBU) (0.188 mmol, 29.1 mg) was subsequently added under argon, and the mixture was stirred for 10 min.

The catalyst solution was transferred to the reactor under argon. The gas phase of the reactor was purged three times with hydrogen and subsequently pressurised with 50 bar of H_2. The catalyst was preformed for 2 h at 160 °C. Shortly before the reaction was started, the oxime (7.5 mmol, 1840.4 mg) was placed in a dried Schlenk flask and dissolved in 10.5 mL of dioxane

under argon counterflow. The resulting solution was transferred to the dropping funnel. To initiate the reaction, the pressure between the reactor and the dropping funnel was first equalised, after which the substrate solution was rapidly introduced into the reactor by opening the valve to the reactor.

During the reaction, samples were withdrawn via a sampling valve and analysed by gas chromatography. Each GC sample consisted of 475 mg of reaction mixture, 25 mg of *n*-decane as internal standard, and 500 mg of isopropanol as solvent. Gas chromatographic analyses were performed using an Agilent 7890A GC equipped with a flame ionisation detector (FID) and an HP-5 column (Agilent Technologies). Further details regarding column specifications and data acquisition are provided in Table S4 and Table S5.

2. Analytics

2.1. Specifications of the column

Table S4: Details of the HP-5 column.

**Aspect**	**Information on the column used**
Manufacturer	Agilent Technologies, Inc.
Product number	19091J-413-INT
Name	HP-5
Permissible temperature range	-60 – 350 °C
Coating material	(5%-Phenyl)-methylpolysiloxane
Film thickness	0.25 µm
Length	30 m
Diameter	320 µm

2.2. Heating Profiles Used

Table S5: Heating profile used in the gas chromatography analysis for the isomerising Methoxycarbonylation, ester reduction, alcohol amination, ester hydrolysis, oxidation and oxime hydrogenation with the HP-5 column.

Heating ramp	Heating rate [°C · min^{-1}]	Temperature [°C]	Holding time [min]	Run time [min]
Start		40	1	1
Ramp 1	40	220	1	6.5
Ramp 2	40	320	3	12

Table S6: Heating profile used in the gas chromatography analysis for the isomerising hydroformylation with the HP-5 column.

Heating ramp	Heating rate [°C · min^{-1}]	Temperature [°C]	Holding time [min]	Run time [min]
Start		60	0	0
Ramp 1	30	110	0	1.67
Ramp 2	1	111.4	3	6.07
Ramp 3	1	115	0	9.67
Ramp 4	30	195	0	12.33
Ramp 5	5	210	0	15.33
Ramp 6	10	250	0	19.33
Ramp 7	1	255	0	24.33
Ramp 8	30	320	0	26.5

2.3. Representative Chromatogram

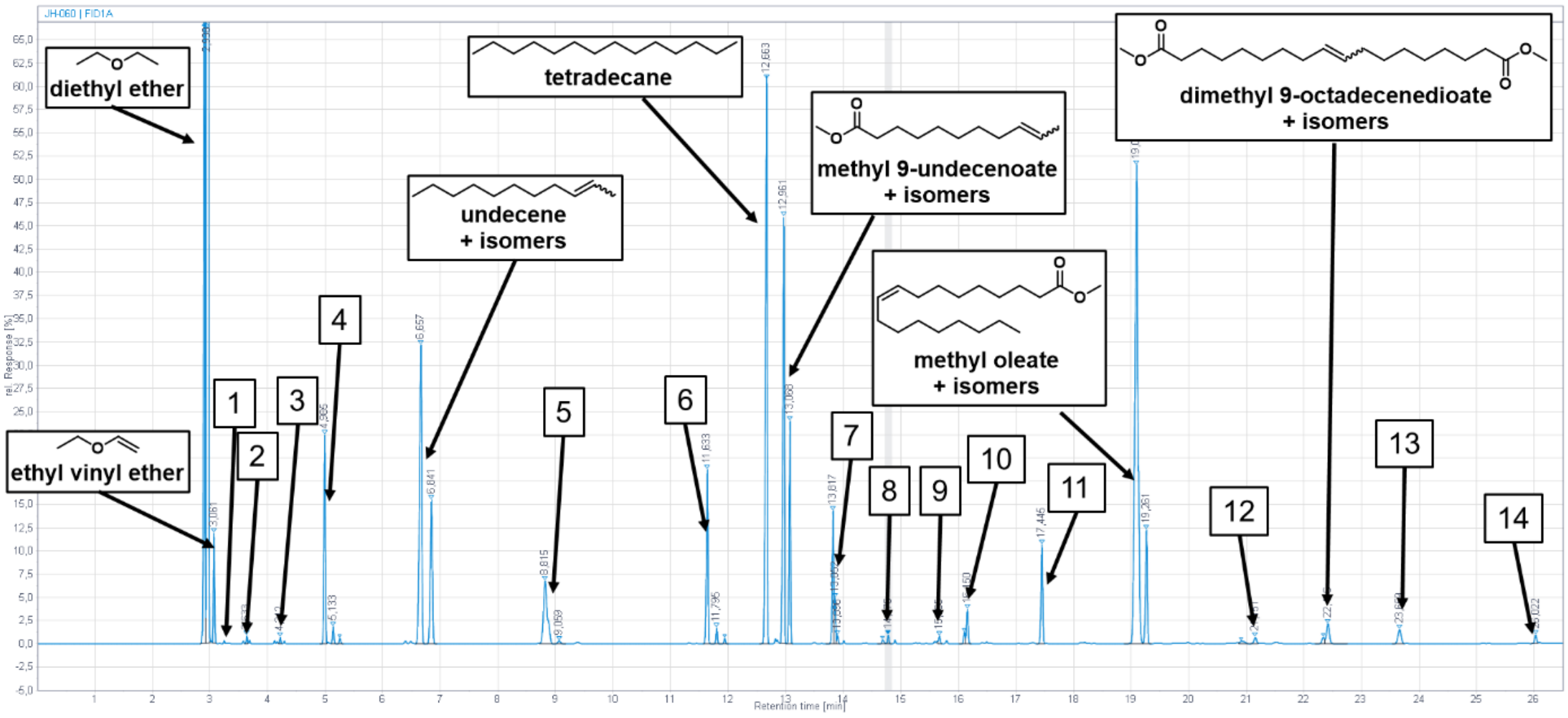

Figure S2: Standard gas chromatogram of the cross-metathesis of methyl oleate and crude 2-butene, showing the products identified using the heating profile presented in Table S6 and tetradecane as internal standard.

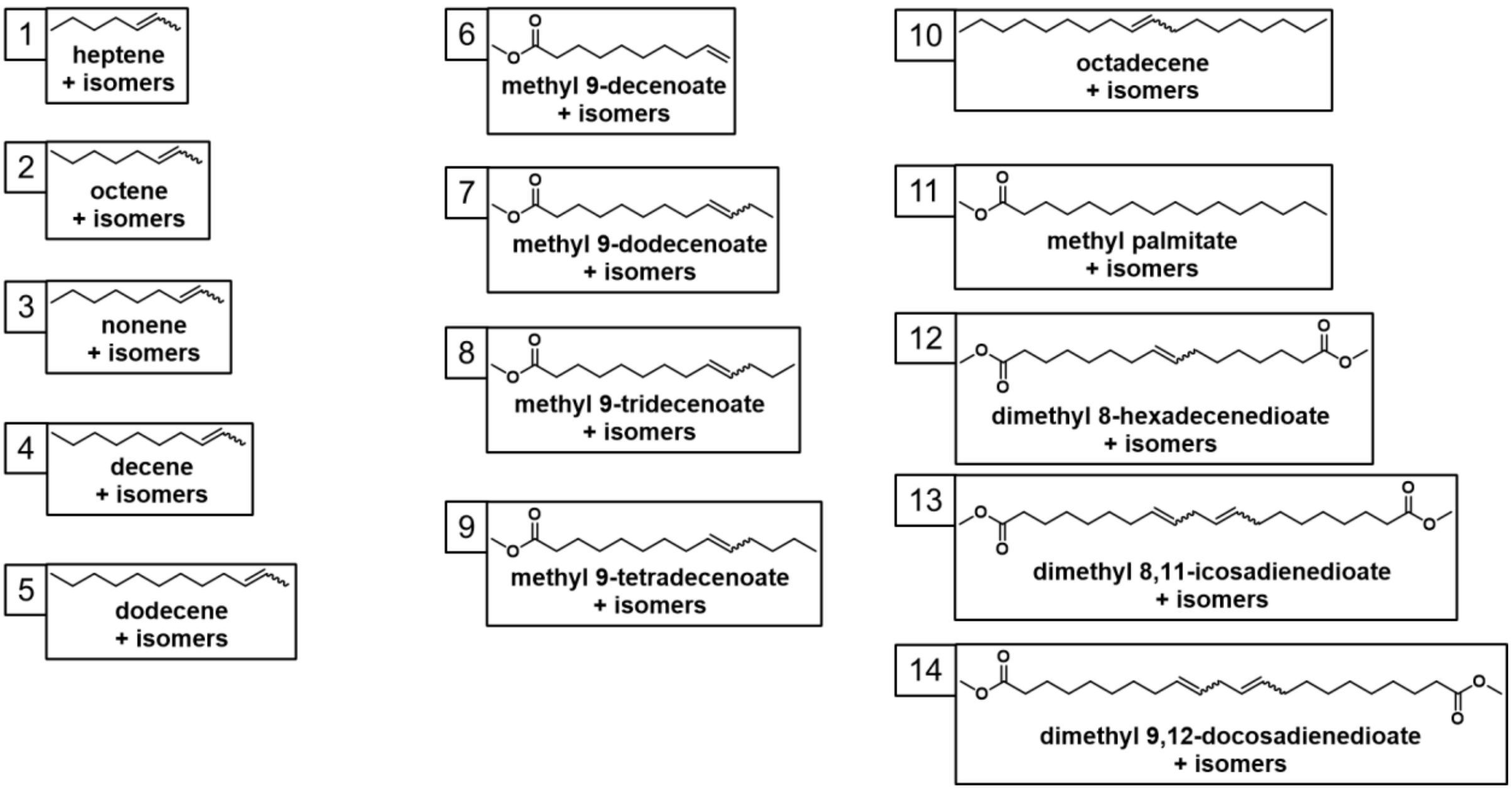

Figure S3: Further listing of products shown in Figure S2.

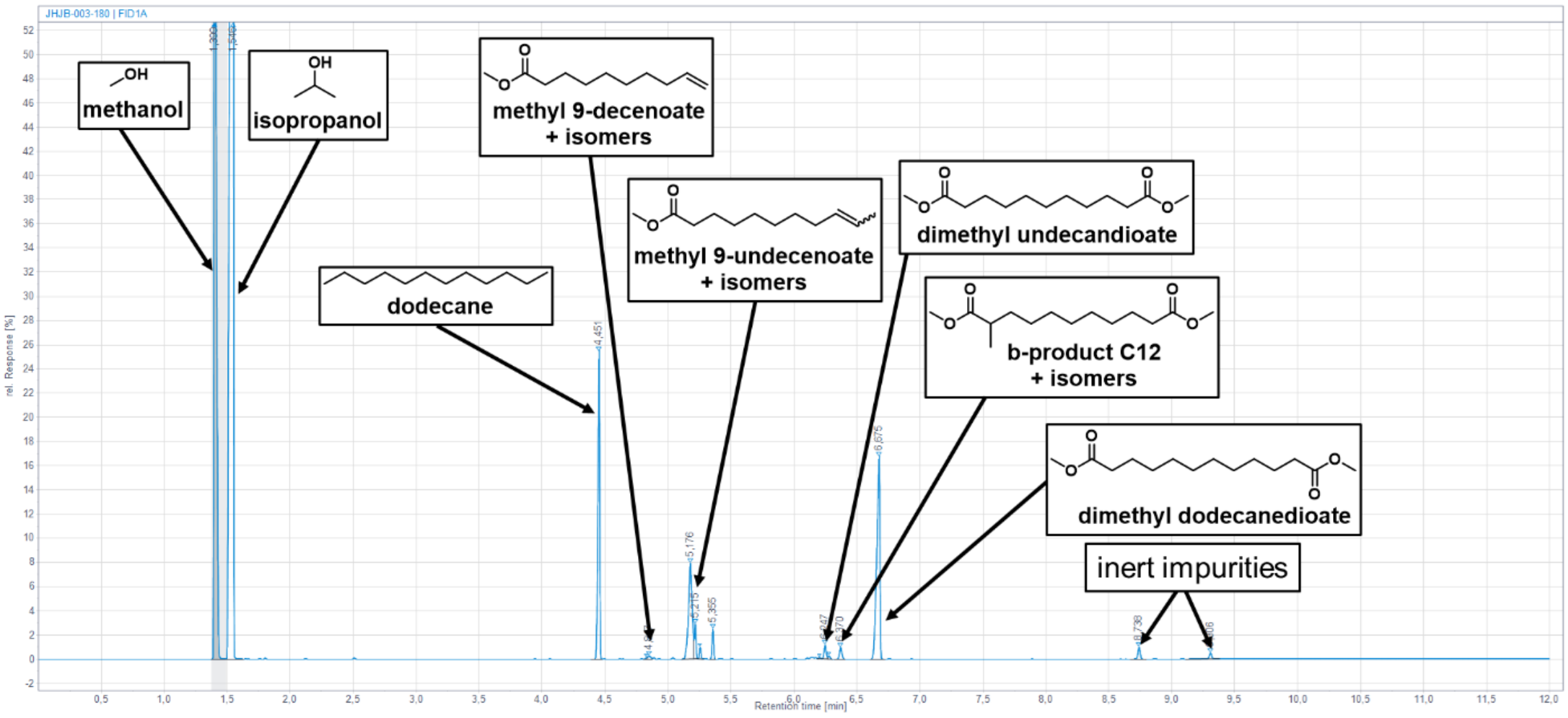

Figure S4: Standard gas chromatogram of the isomerising methoxycarbonylation of methyl 9-undecenoate, showing the products identified using the heating profile presented in Table S5 and *n*-dodecane as internal standard.

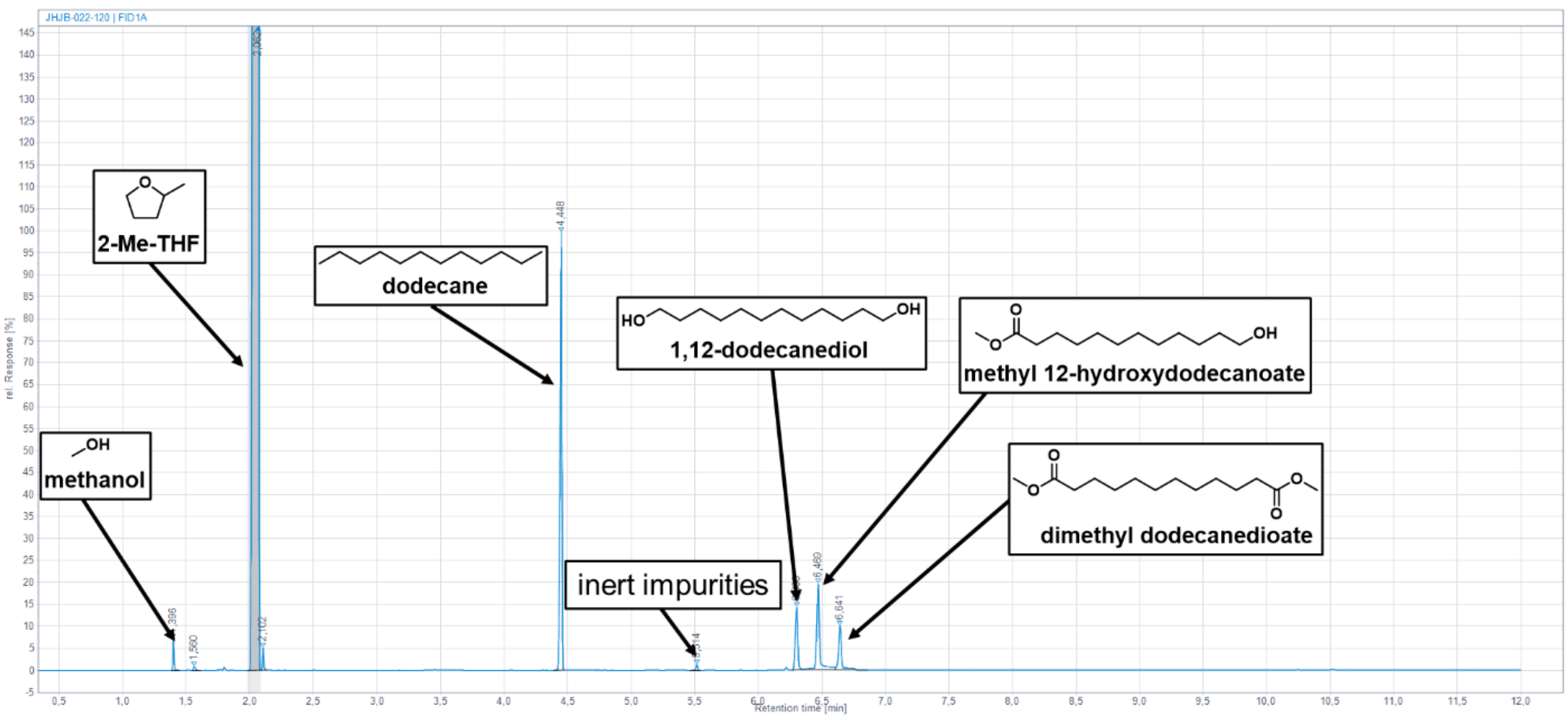

Figure S5: Standard gas chromatogram of the ester-reduction of dimethyl dodecanedioate, showing the products identified using the heating profile presented in Table S5 and *n*-dodecane as internal standard.

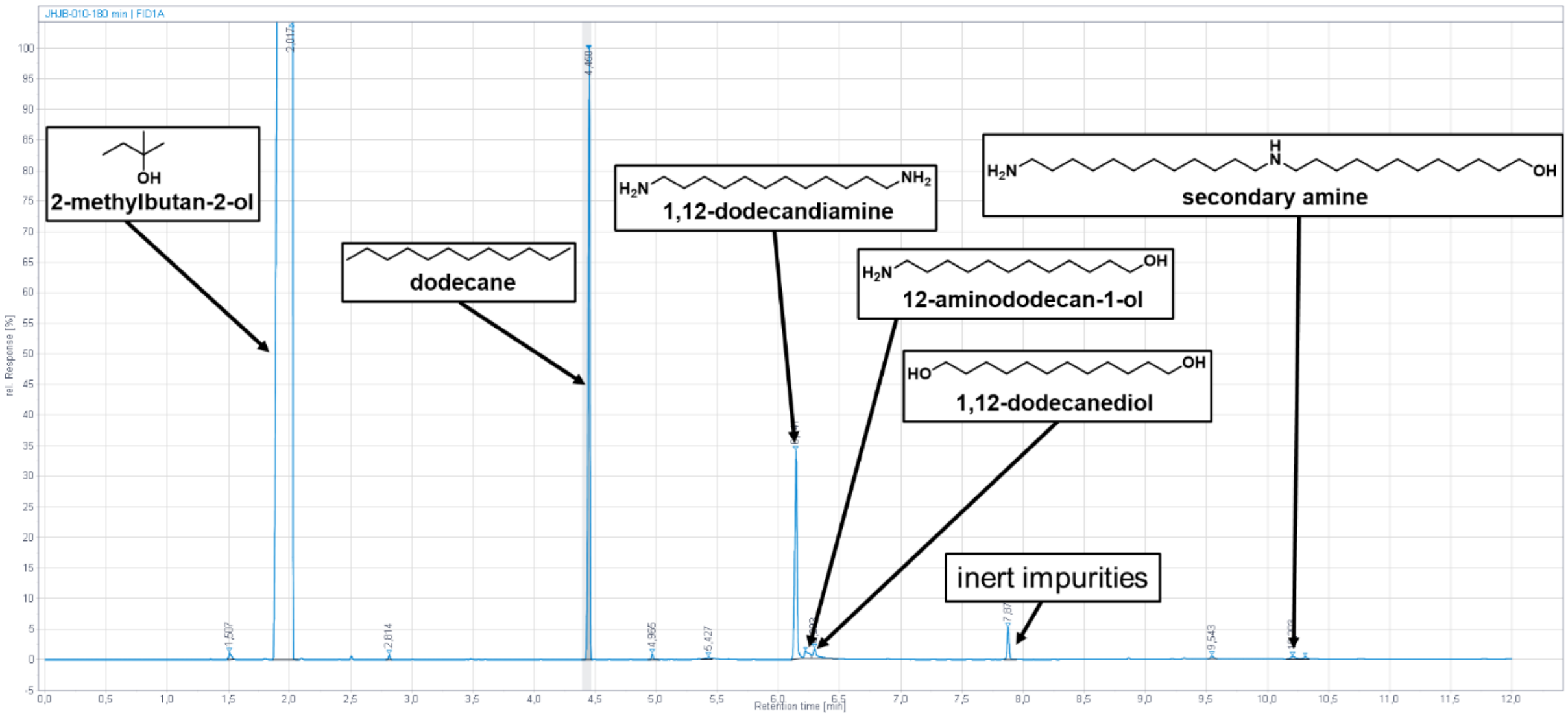

Figure S6: Standard gas chromatogram of the alcohol amination of 1,12-dodecandiol, showing the products identified using the heating profile presented in Table S5 and *n*-dodecane as internal standard.

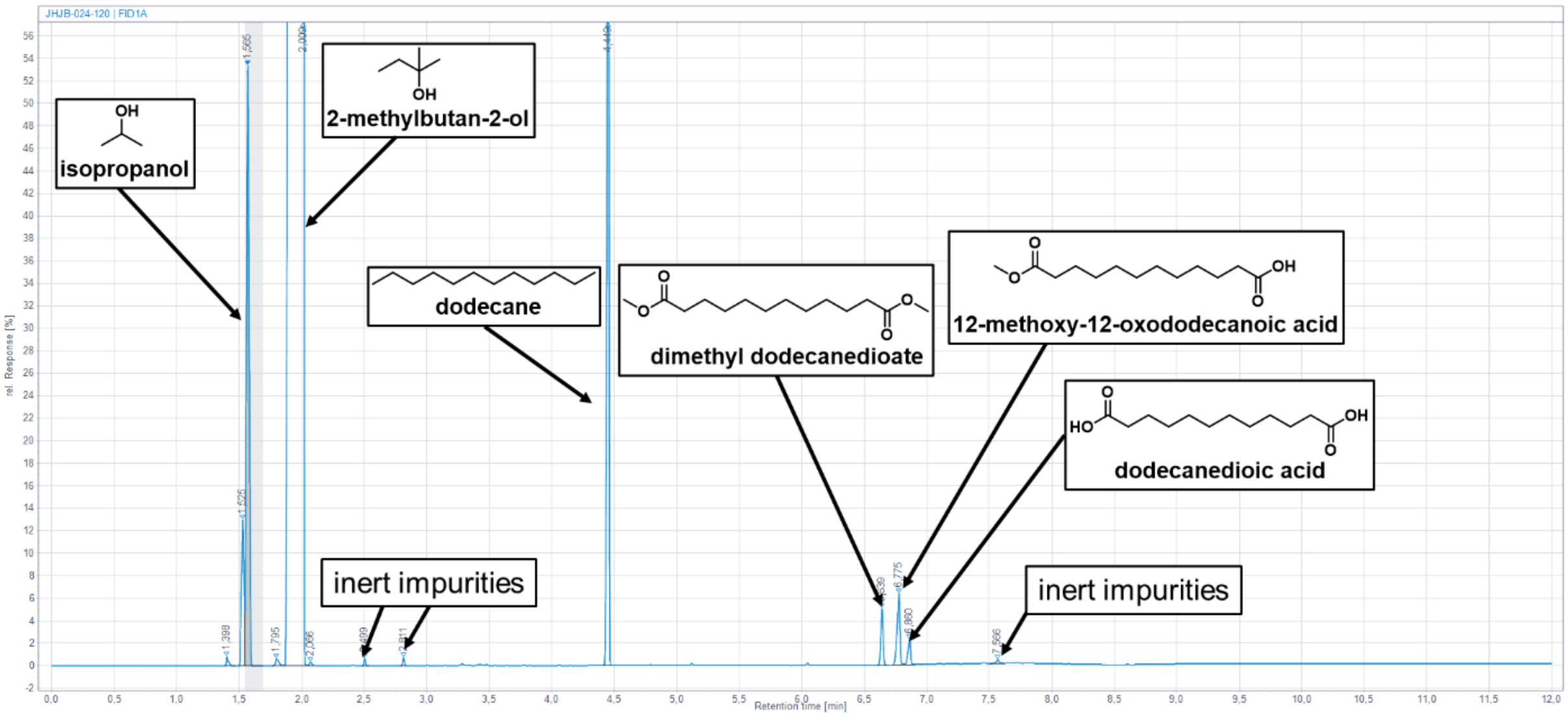

Figure S7: Standard gas chromatogram of the hydrolysis of dimethyl dodecandioate, showing the products identified using the heating profile presented in Table S5 and *n*-dodecane as internal standard.

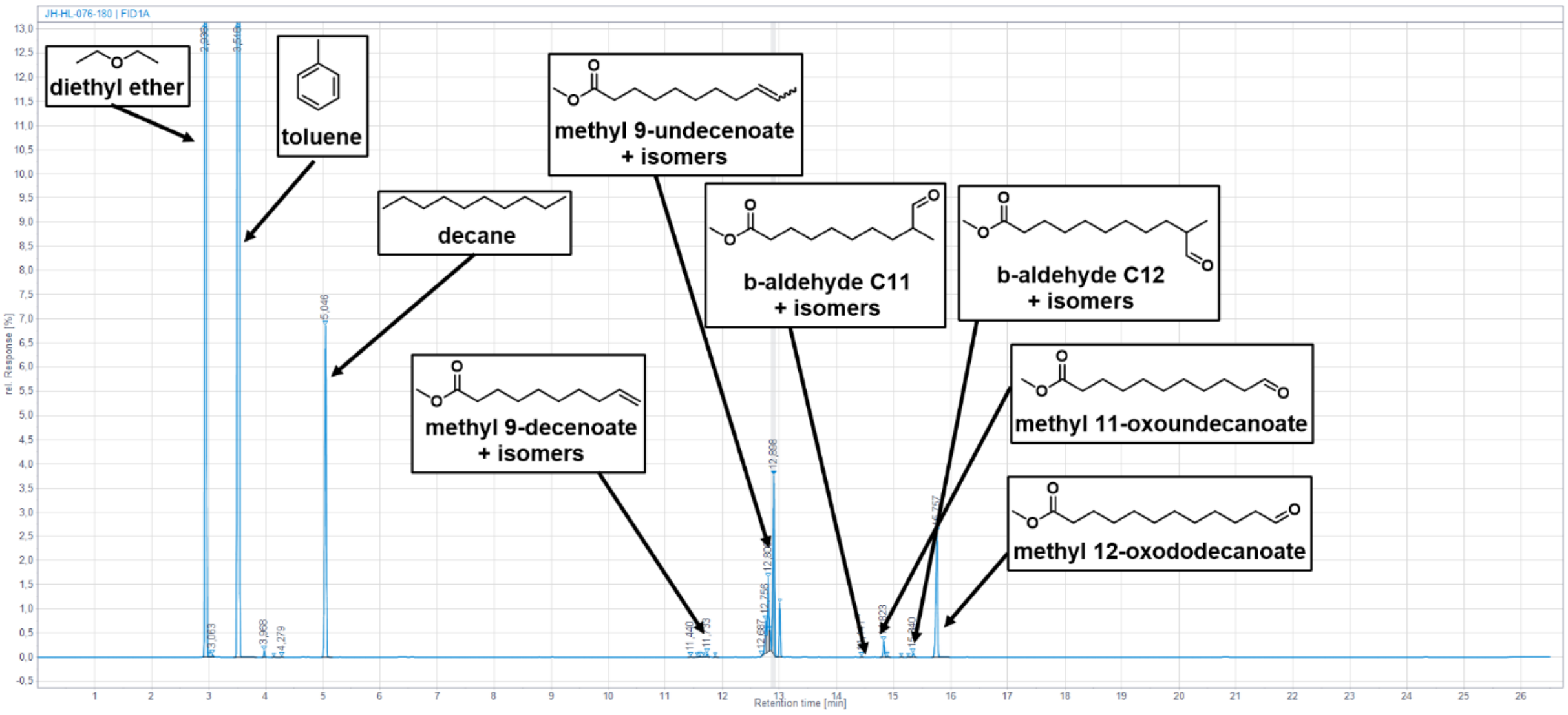

Figure S8: Standard gas chromatogram of the isomerising hydroformylation of methyl 9-undecenoate, showing the products identified using the heating profile presented in Table S6 and *n*-decane as internal standard.

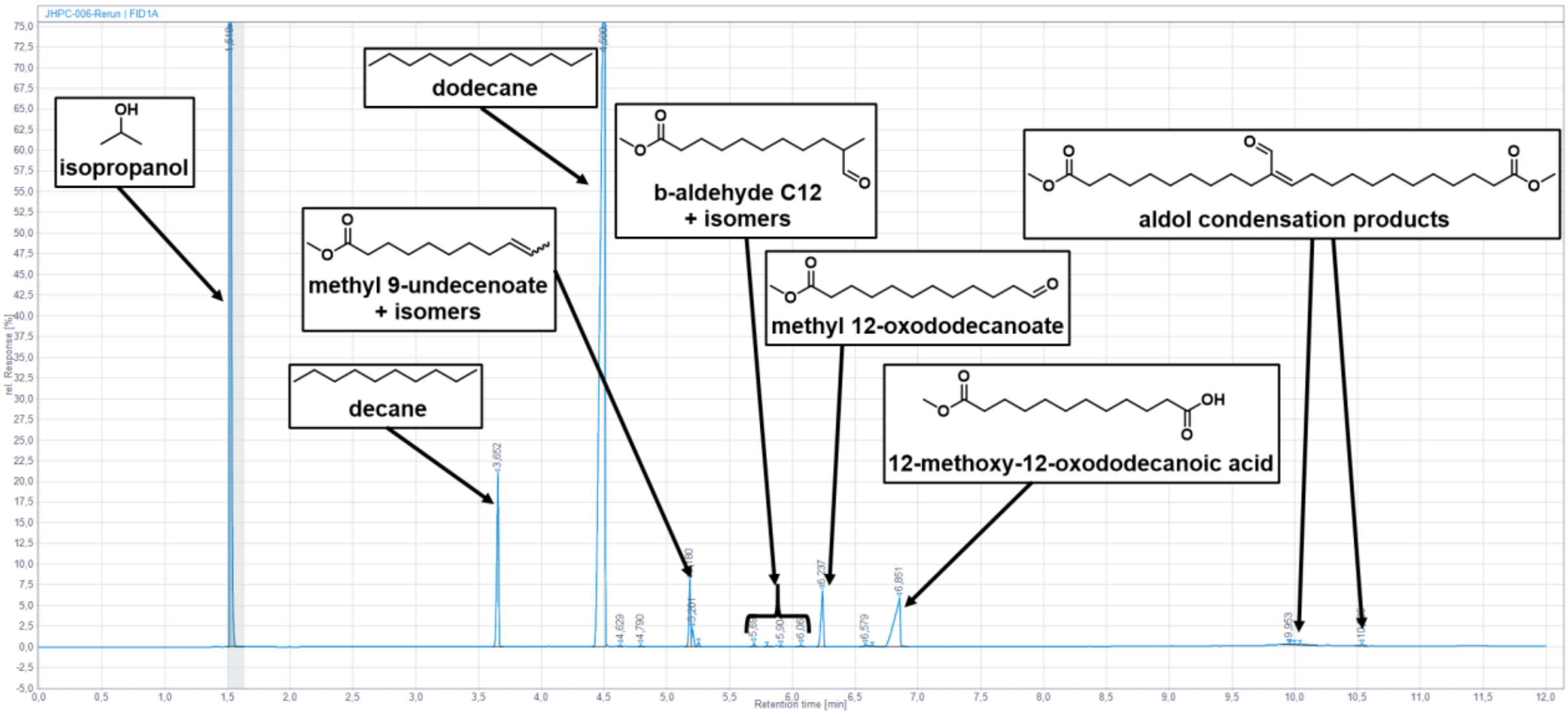

Figure S9: Standard gas chromatogram of the oxidation of methyl 12-oxododecanoate, showing the products identified using the heating profile presented in Table S5 and *n*-decane as internal standard.

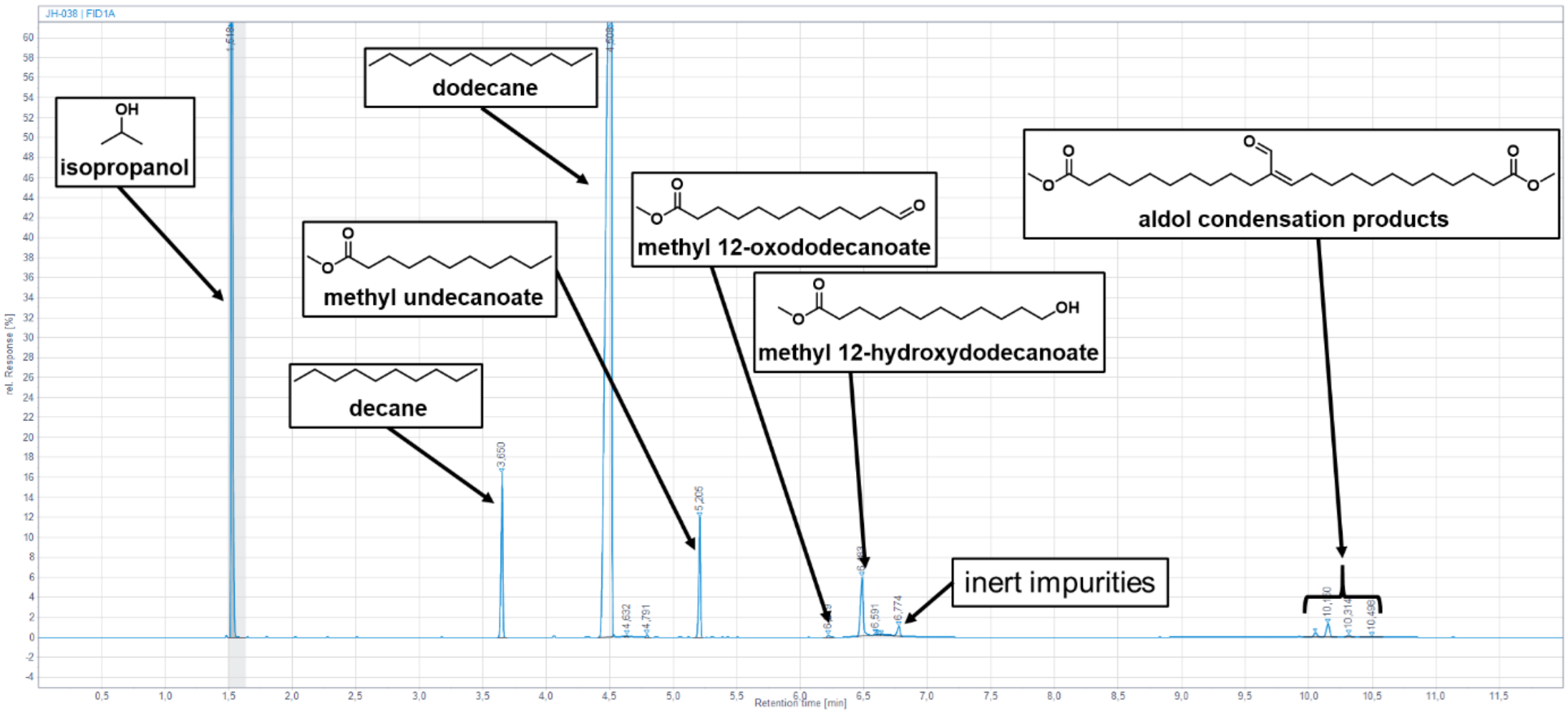

Figure S10: Standard gas chromatogram of the hydrogenation of methyl 12-oxododecanoate, showing the products identified using the heating profile presented in Table S5 and *n*-decane as internal standard.

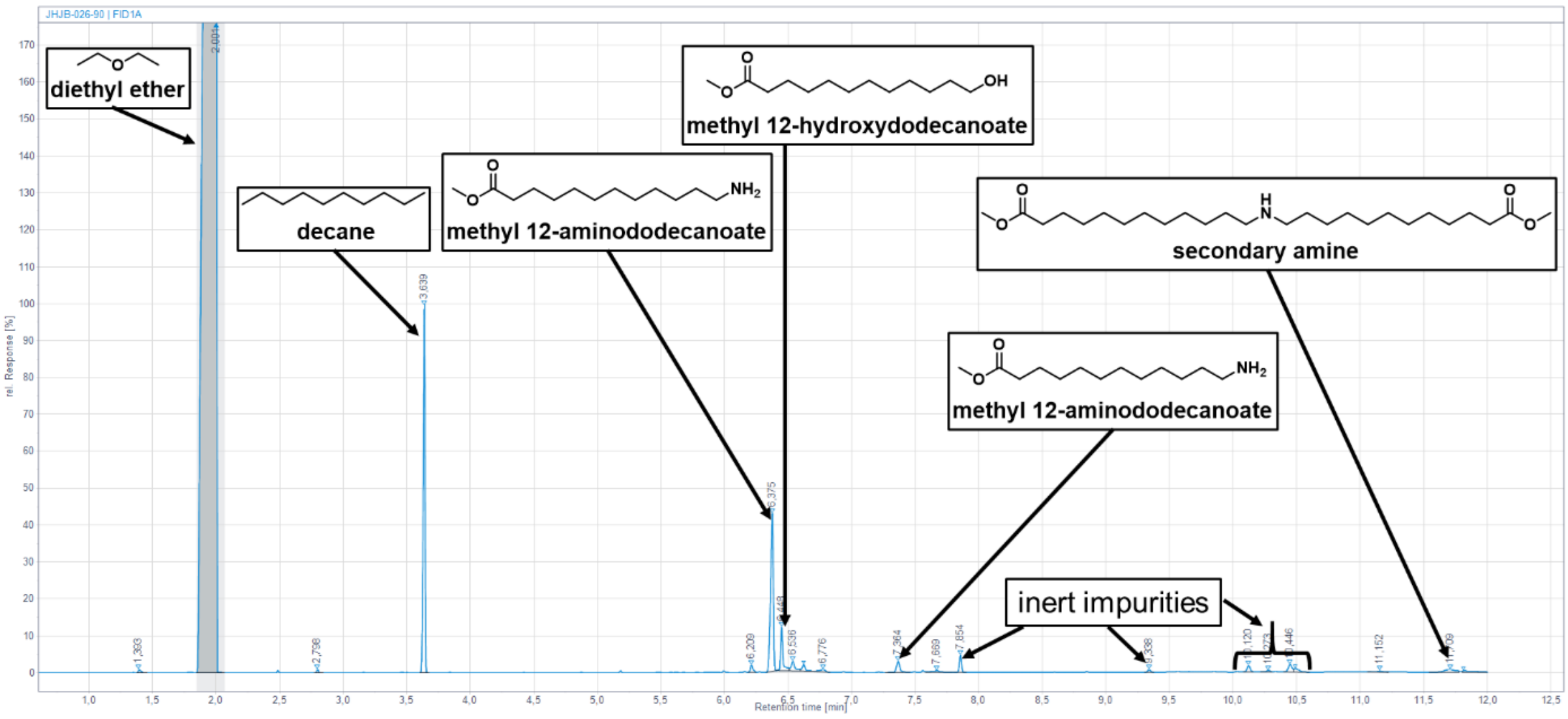

Figure S11: Standard gas chromatogram of the alcohol amination of methyl 12-hydroxydodecanoate, showing the products identified using the heating profile presented in Table S5 and *n*-decane as internal standard

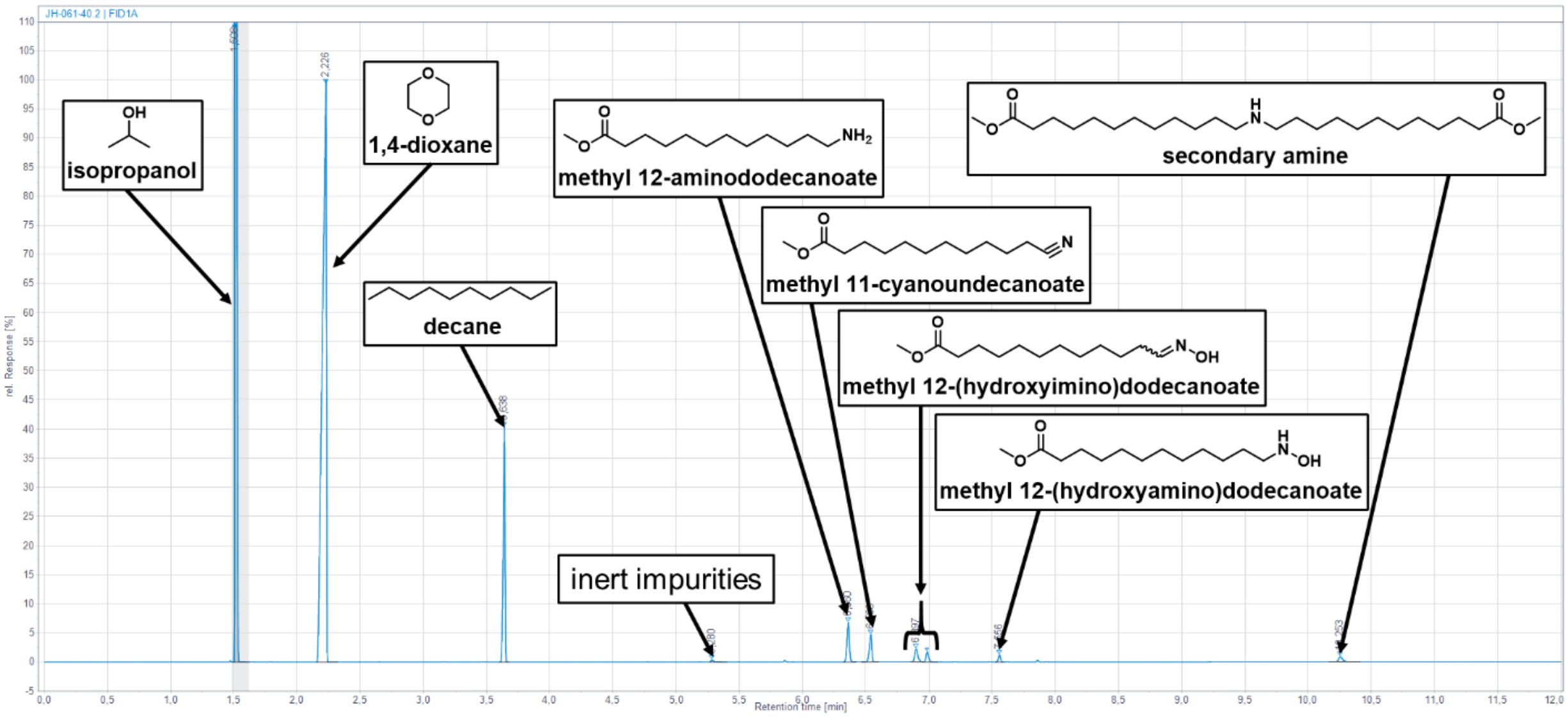

Figure S12: Standard gas chromatogram of the hydrogenation of the oxime, methyl 12-(hydroxyamino)dodecanoate, showing the products identified using the heating profile presented in Table S5 and *n*-decane as internal standard.

3. Additional Results

3.1. Cross-Metathesis Optimisation

Conditions temperature screening: 11.7 mmol of methyl oleate (soybean oil methyl ester, semi-hydrogenated, corresponding to the amount of double bonds), 1000 ppm UltraCat, methyl oleate to 2-butene ratio of 1:1, *T* = 20 – 100 °C, *t* = 3 h, 500 rpm.

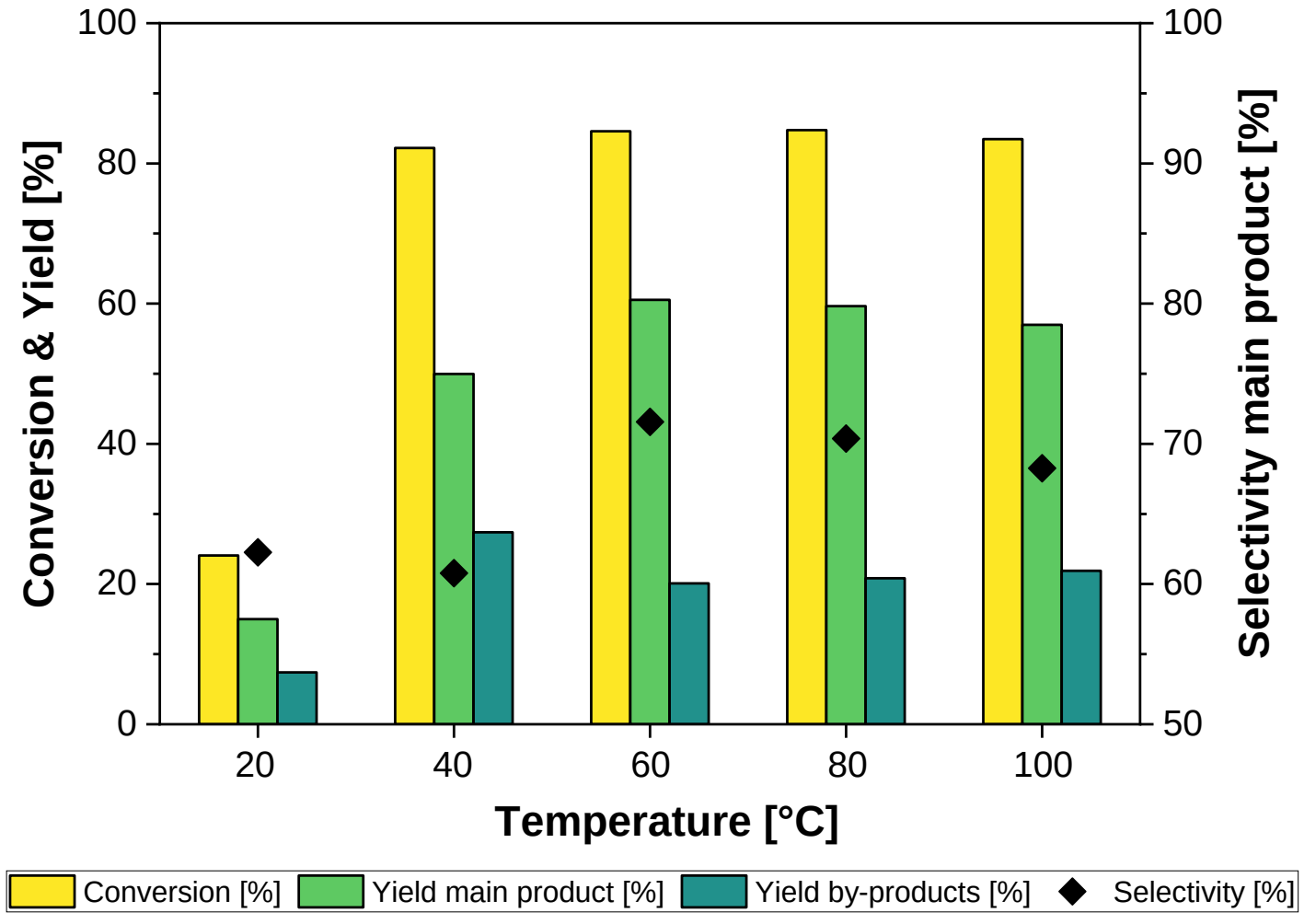

Figure S13: Results of the temperature screening for the cross-metathesis of soybean oil methyl ester with 2-butene. All yields and conversions were determined *via* GC-FID with the internal standard *n*-tetradecane.

Conditions catalyst loading: 11.7 mmol of methyl oleate (soybean oil methyl ester, semi-hydrogenated, corresponding to the amount of double bonds), 250 - 5000 ppm UltraCat, methyl oleate to 2-butene ratio of 1:1, *T* = 80 °C, *t* = 3 h, 500 rpm.

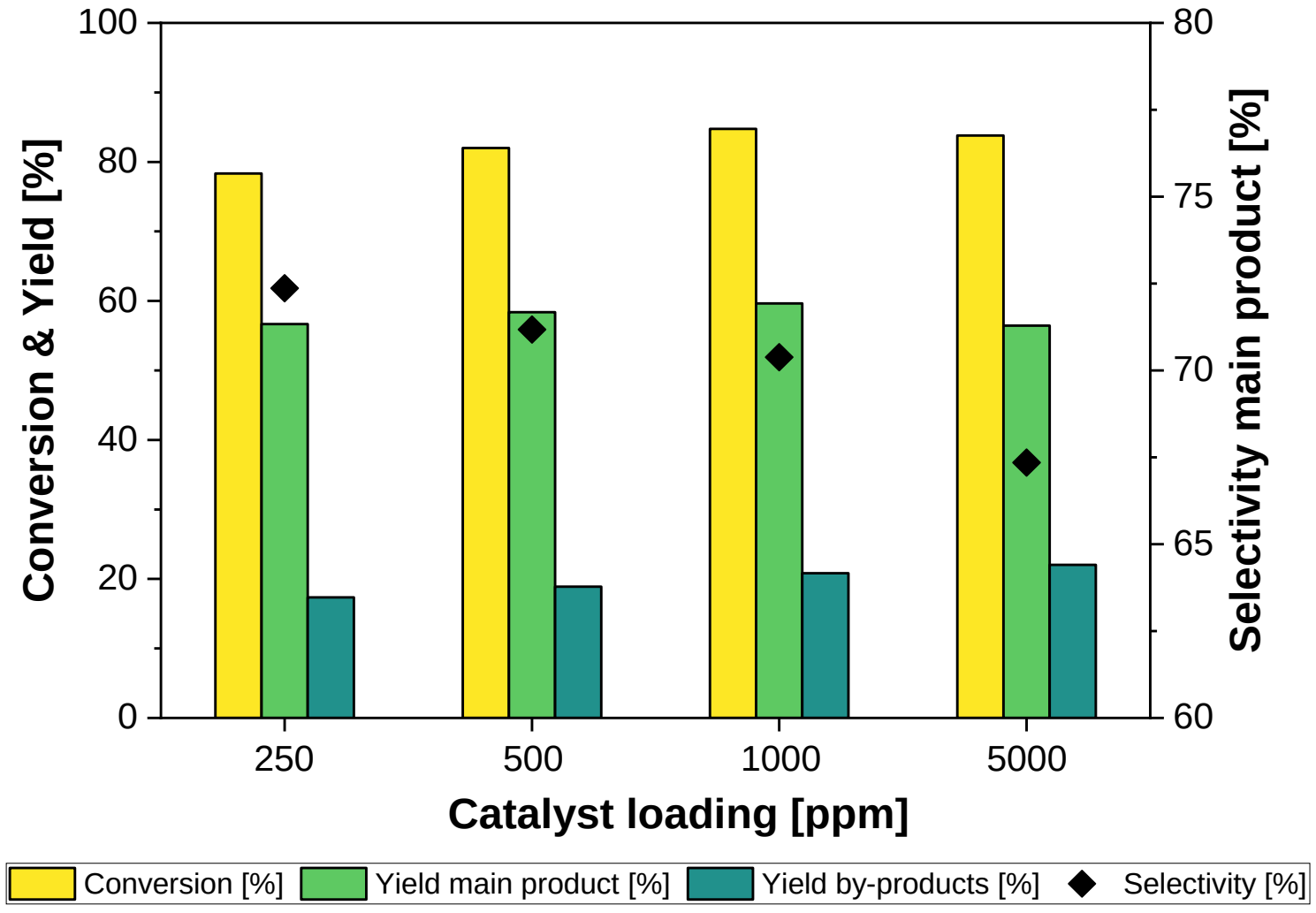

Figure S14: Results of the variation of the catalyst loading for the cross-metathesis of soybean oil methyl ester with 2-butene. All yields and conversions were determined *via* GC-FID with the internal standard *n*-tetradecane.

Conditions: 11.7 mmol of methyl oleate (soybean oil methyl ester, semi-hydrogenated, corresponding to the amount of double bonds), 250 ppm UltraCat, methyl oleate to 2-butene ratio of 1:1 to 1:3, *T* = 80 °C, *t* = 3 h, 500 rpm.

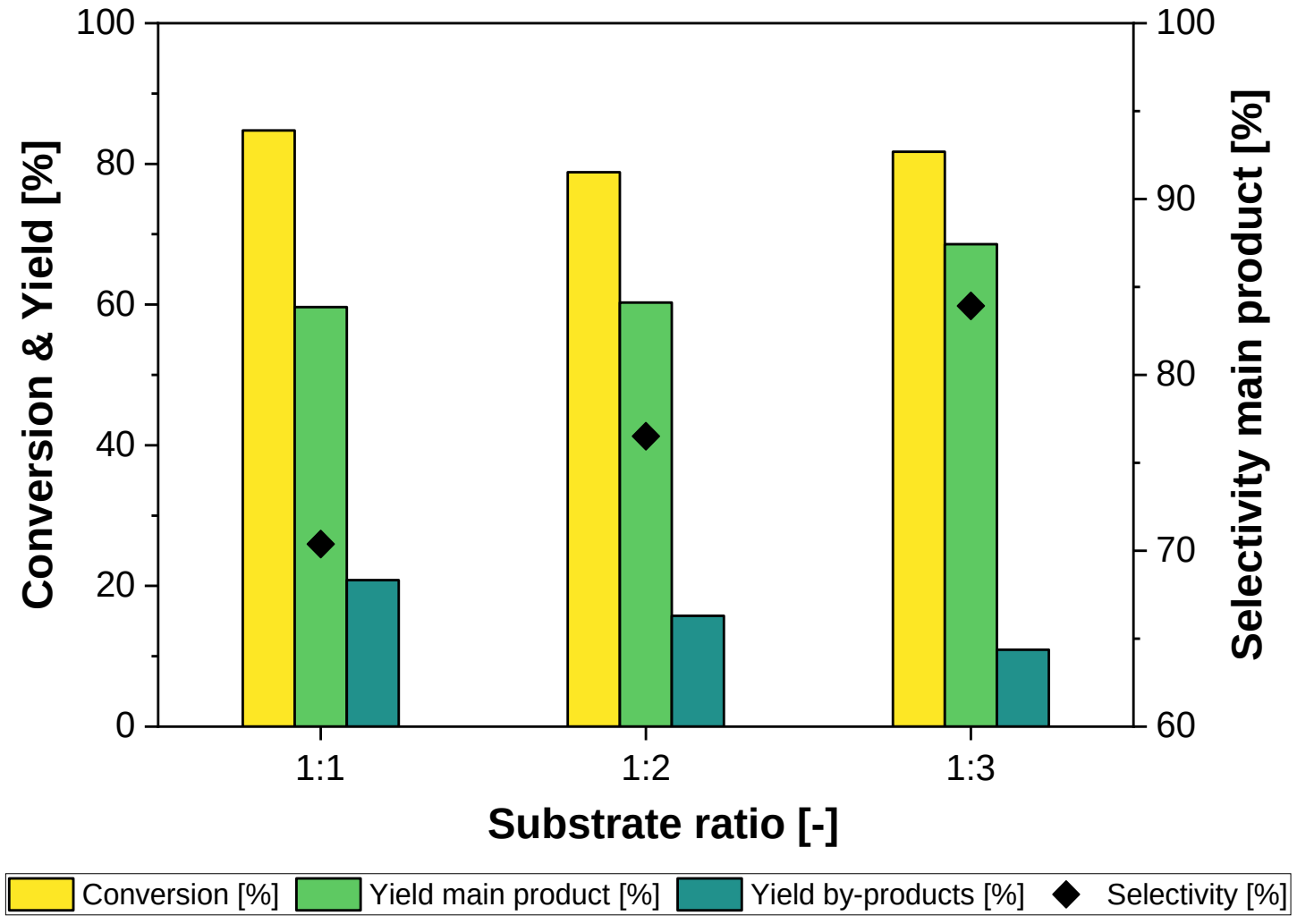

Figure S15: Results of the ratio variation of soybean oil methyl ester and 2-butene from 1:1 to 1:3. All yields and conversions were determined *via* GC-FID with the internal standard *n*-tetradecane.

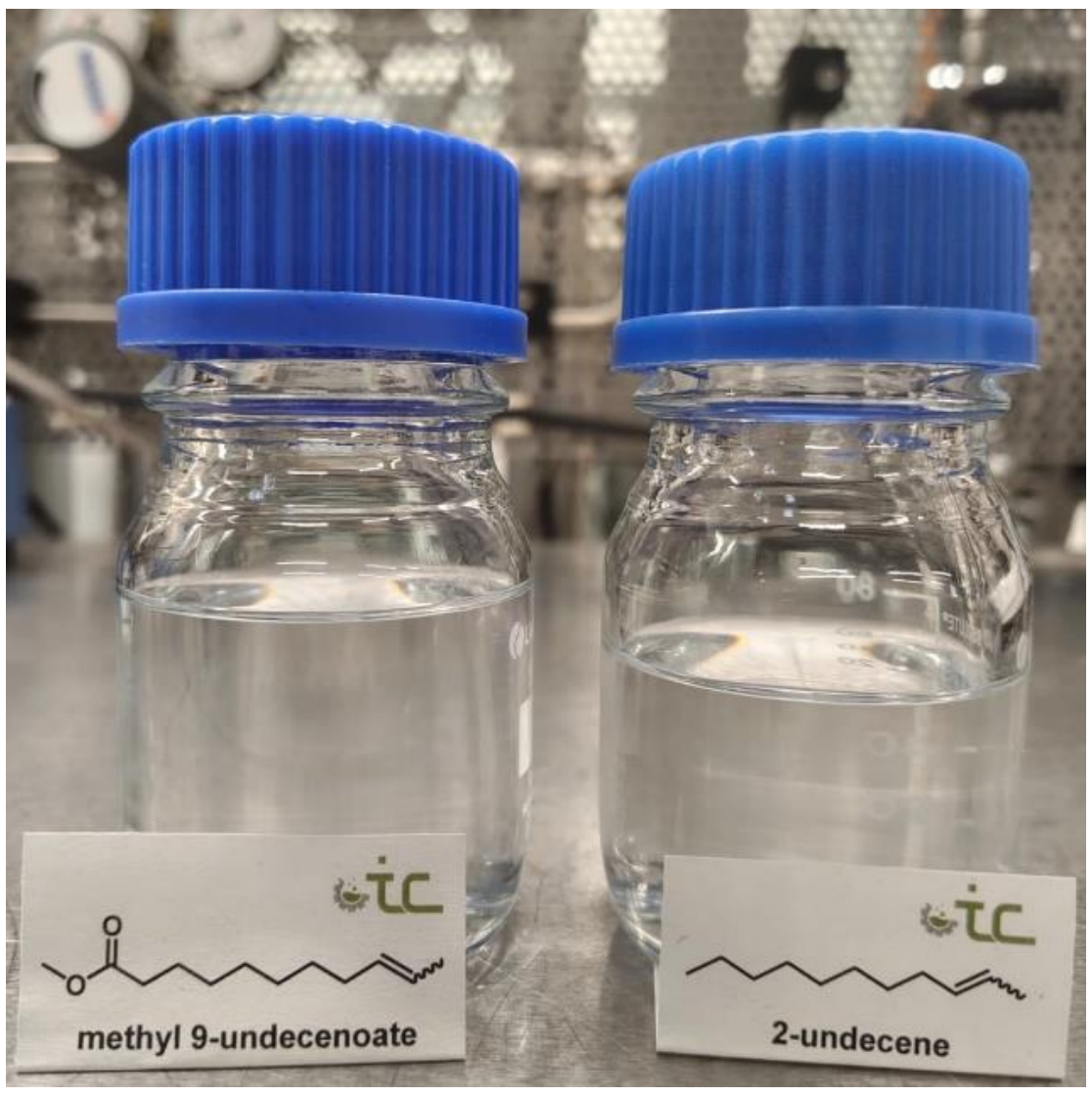

Figure S16: Highly pure methyl 9-undecenoate and 2-undecene obtained by distillation of the crude cross-metathesis reaction mixture.

3.2. Isomerising Methoxycarbonylation

Conditions: 16.9 mmol methyl 9-undecenoate, 0.05 mol% (8.5 * 10^{-3} mmol) Pd_2dba_3, 1.75 mol% (0.3 mmol) 1,2-DTBPMB, 30 mL MeOH, p_{CO} = 30 bar, T = 95 °C, t = 23 h, 1000 rpm.

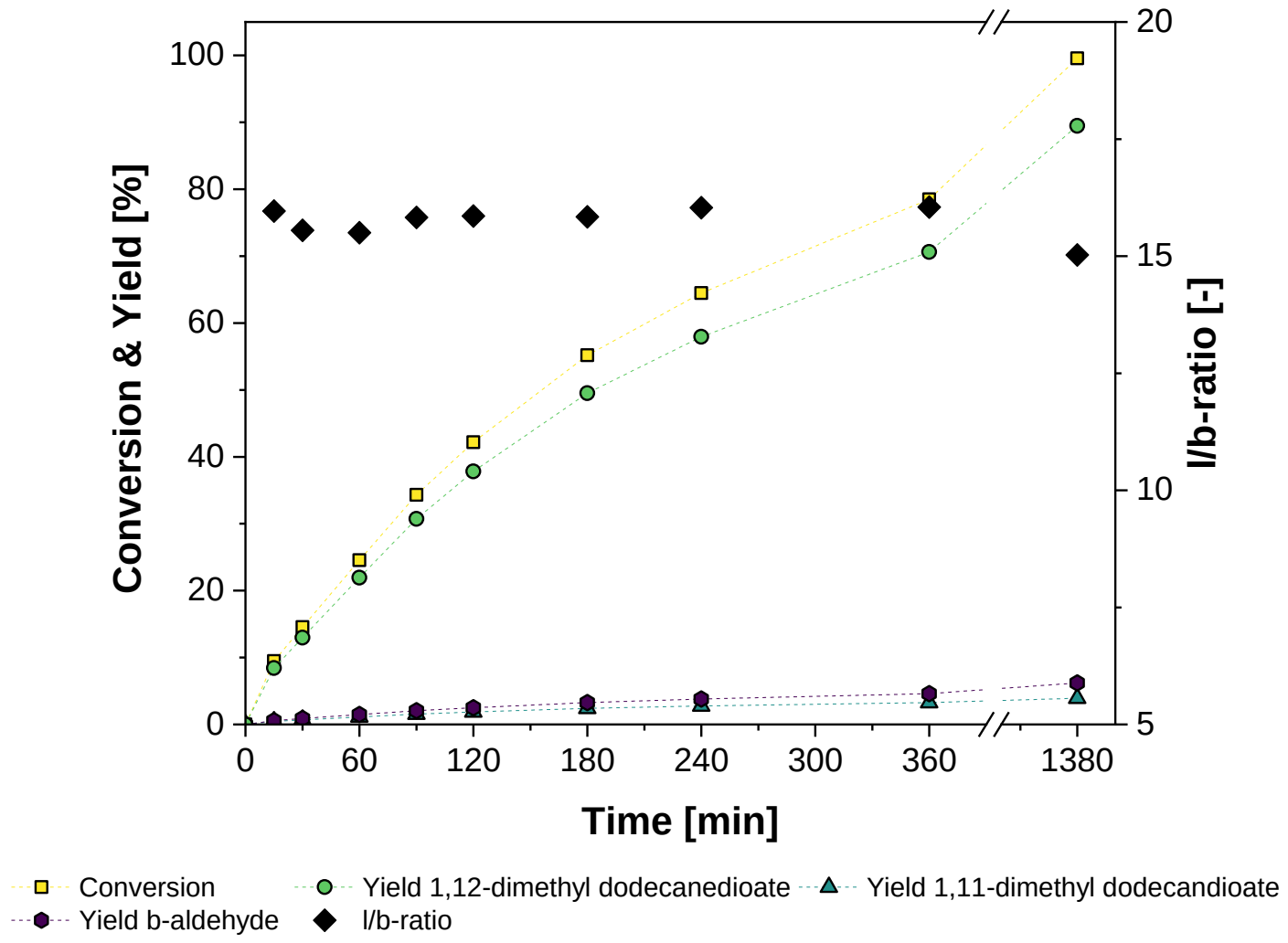

Figure S17: Reaction profile of the isomerising Methoxycarbonylation of methyl 9-undecenoate. All yields and conversions were determined *via* GC-FID with the internal standard *n*-dodecane.

3.3. Alcohol Amination of 1,12-dodecandiol

Conditions: 7.5 mmol 1,12-dodecanediol, 0.83 mol% ($6.2 * 10^{-2}$ mmol) $HRuCl(CO)(PPh_3)_3$, 1.66 mol% ($1.2 * 10^{-1}$ mmol) xantphos, 37.5 mL *tert*-amyl alcohol, p_{argon} = 15 bar, T = 170 °C, t = 3 h, 1000 rpm.

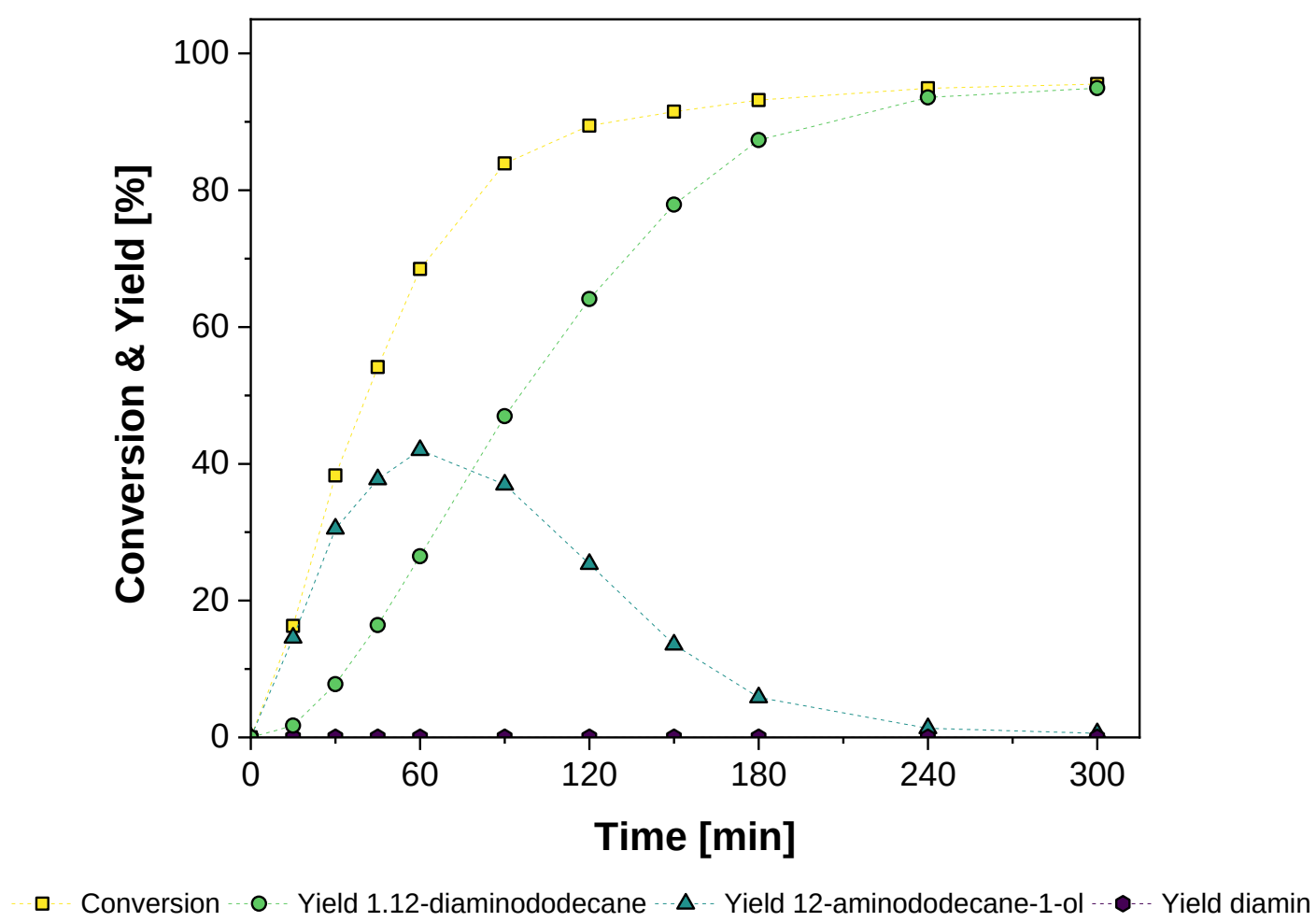

Figure S18: Reaction profile of the alcohol amination of 1,12-dodecandiol. All yields and conversions were determined *via* GC-FID with the internal standard *n*-dodecane.

3.4. Isomerising Hydroformylation

Conditions: 20.8 mmol methyl 9-undecenoate, 0.47 mol% ($9.7 * 10^{-2}$ mmol) Rh(acac)(COD), 1.88 mol% ($3.9 * 10^{-1}$ mmol) BiPhePhos, 40 mL toluene, $p_{CO/H2}$ = 12 bar, T = 118 °C, t = 3 h, 1000 rpm.

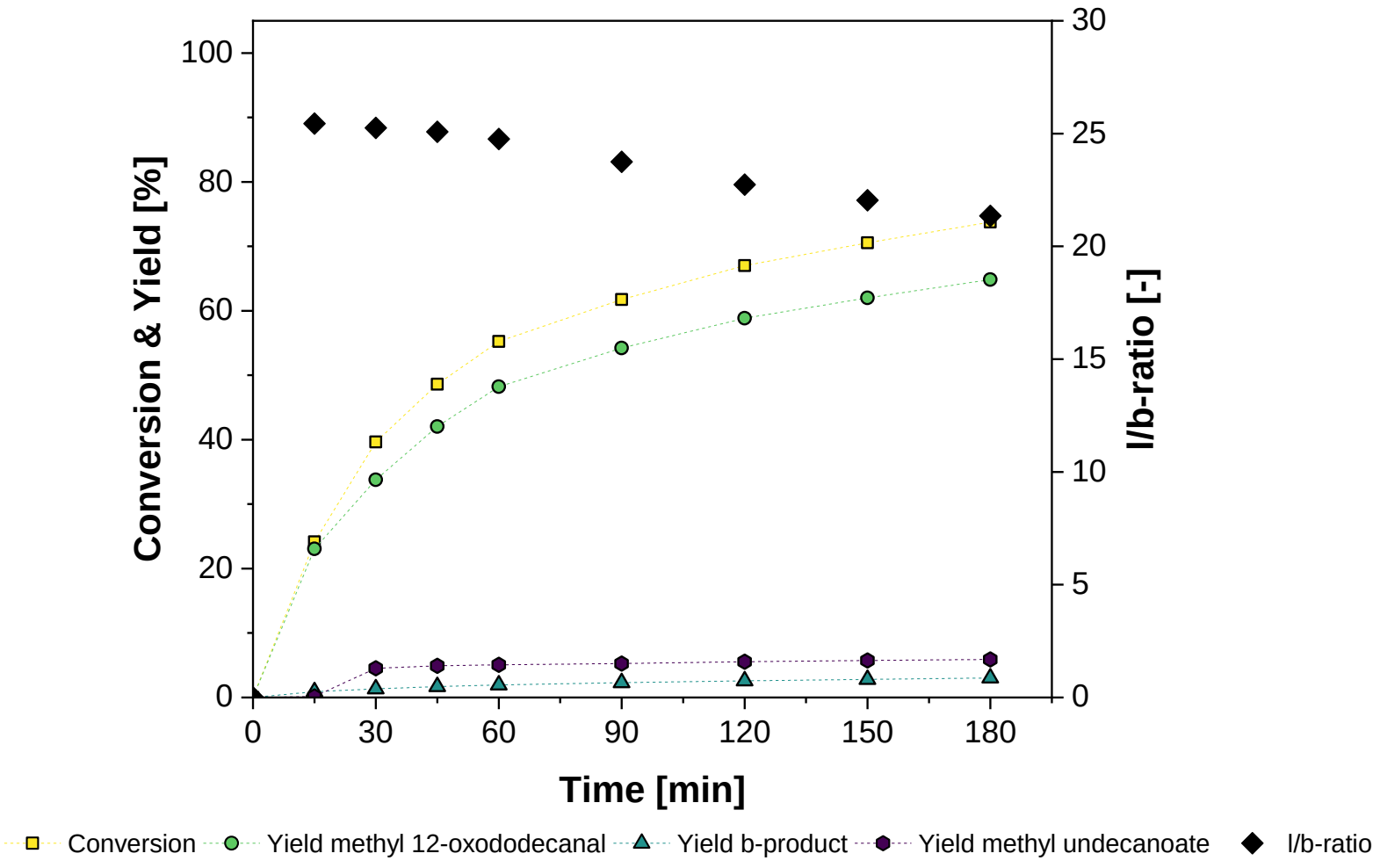

Figure S19: Reaction profile of the isomerising hydroformylation using DoE-optimised reaction parameters. All yields and conversions were determined *via* GC-FID with the internal standard *n*-decane.

Conditions: 20.8 mmol methyl 9-undecenoate, 0.1 mol% (2.1 * 10^{-2} mmol) Rh(acac)(COD), 0.4 mol% (8.3 * 10^{-2} mmol) BiPhePhos, 40 mL toluene, $p_{CO/H2}$ = 5 bar, T = 105 °C, t = 22 h, 1000 rpm.

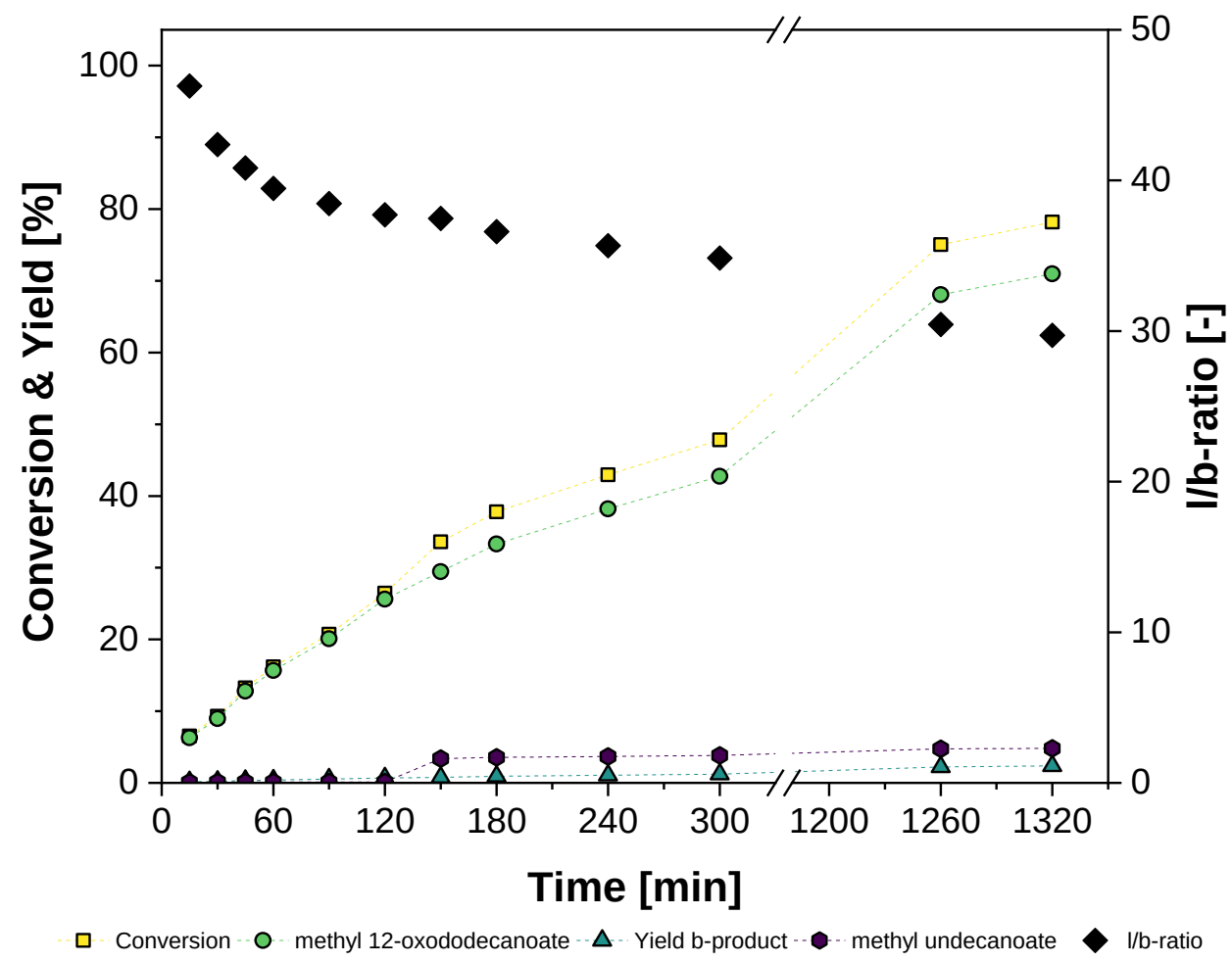

Figure S20: Reaction profile of the isomerising hydroformylation using modified parameters designed to further increase selectivity. All yields and conversions were determined *via* GC-FID with the internal standard *n*-decane.

3.5. Alcohol Amination of methyl 12-hydroxydodecanoate

Conditions: 15 mmol methyl 12-hydroxydodecanoate, 1.67 mo% (1.3 * 10^{-1} mmol) $RuHCl(CO)(PPh_3)_3$, 3.32 mol% (2.5 * 10^{-1} mmol) xantphos, 37.5 mL *tert*-amyl alcohol, p_{argon} = 15 bar, T = 170 °C, t = 3 h, 1000 rpm.

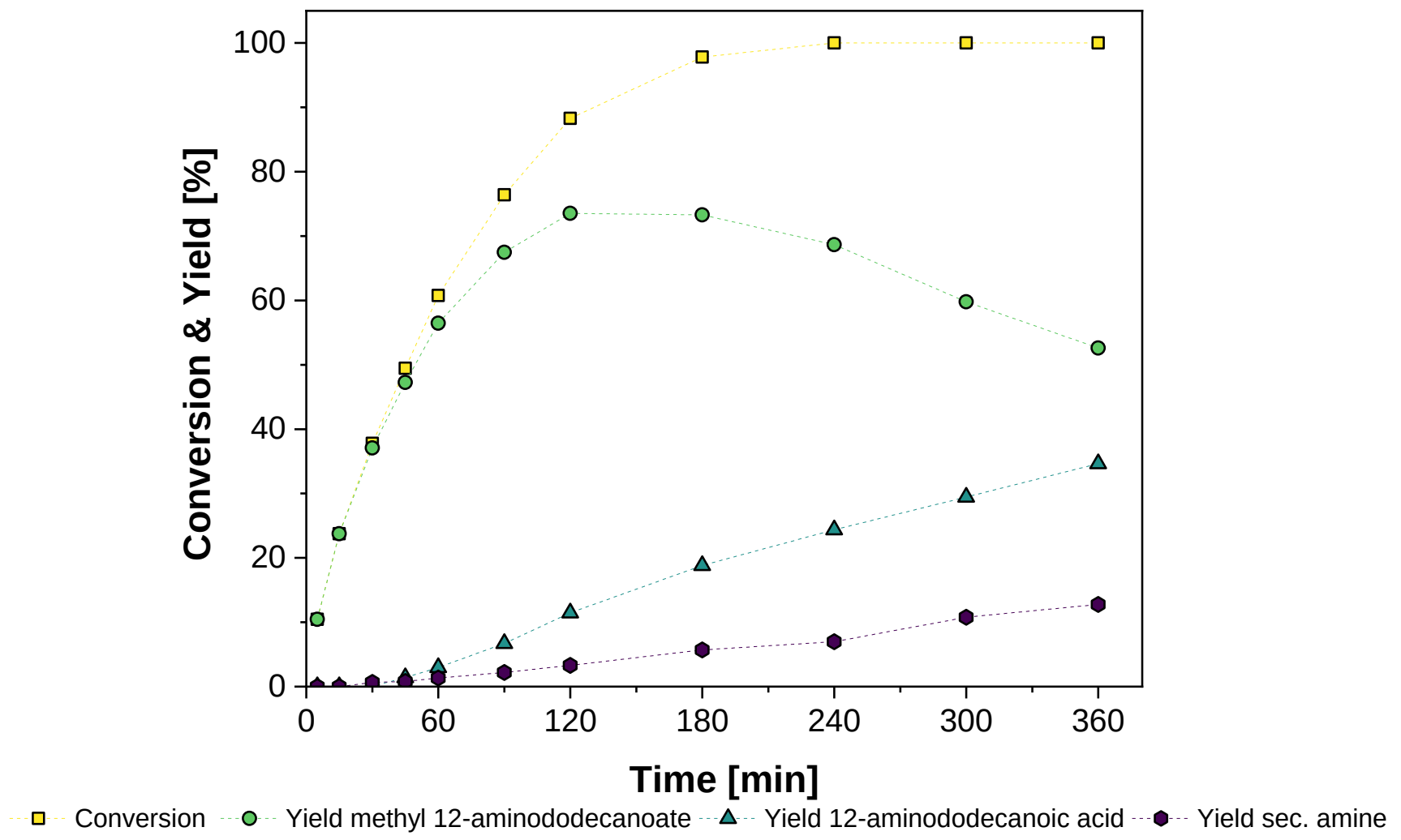

Figure S21: Reaction profile of the alcohol amination of methyl 12-hydroxydodecanoat. All yields and conversions were determined *via* GC-FID with the internal standard *n*-decane.

3.6. Oxime Hydrogenation

Conditions: 7.5 mmol methyl 12-(hydroxyamino)dodecanoate, 0.5 mol% (3.8 * 10^{-2} mmol) $Ru(acac)_3$, 0.55 mol% (4.1 * 10^{-2} mmol) triphos, 2.5 mol% (1.9 * 10^{-1} mmol) DBU, 30 mL dioxane, p_{H2} = 50 bar, T = 160 °C, t = 1 h, 1000 rpm.

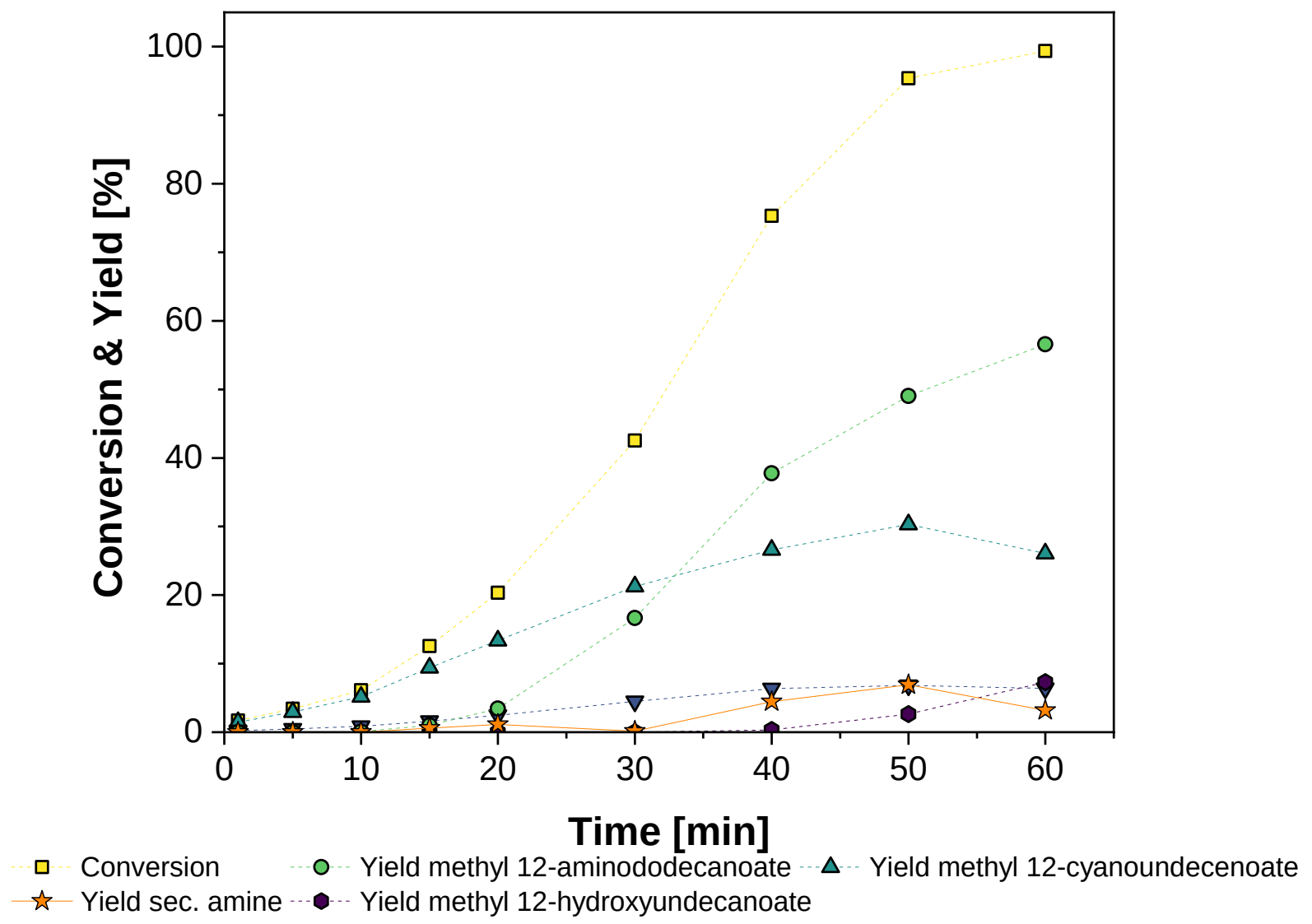

Figure S22: Reaction profile of the oxime hydrogenation of methyl 12-(hydroxyimino)dodecanoate. All yields and conversions were determined *via* GC-FID with the internal standard *n*-decane.

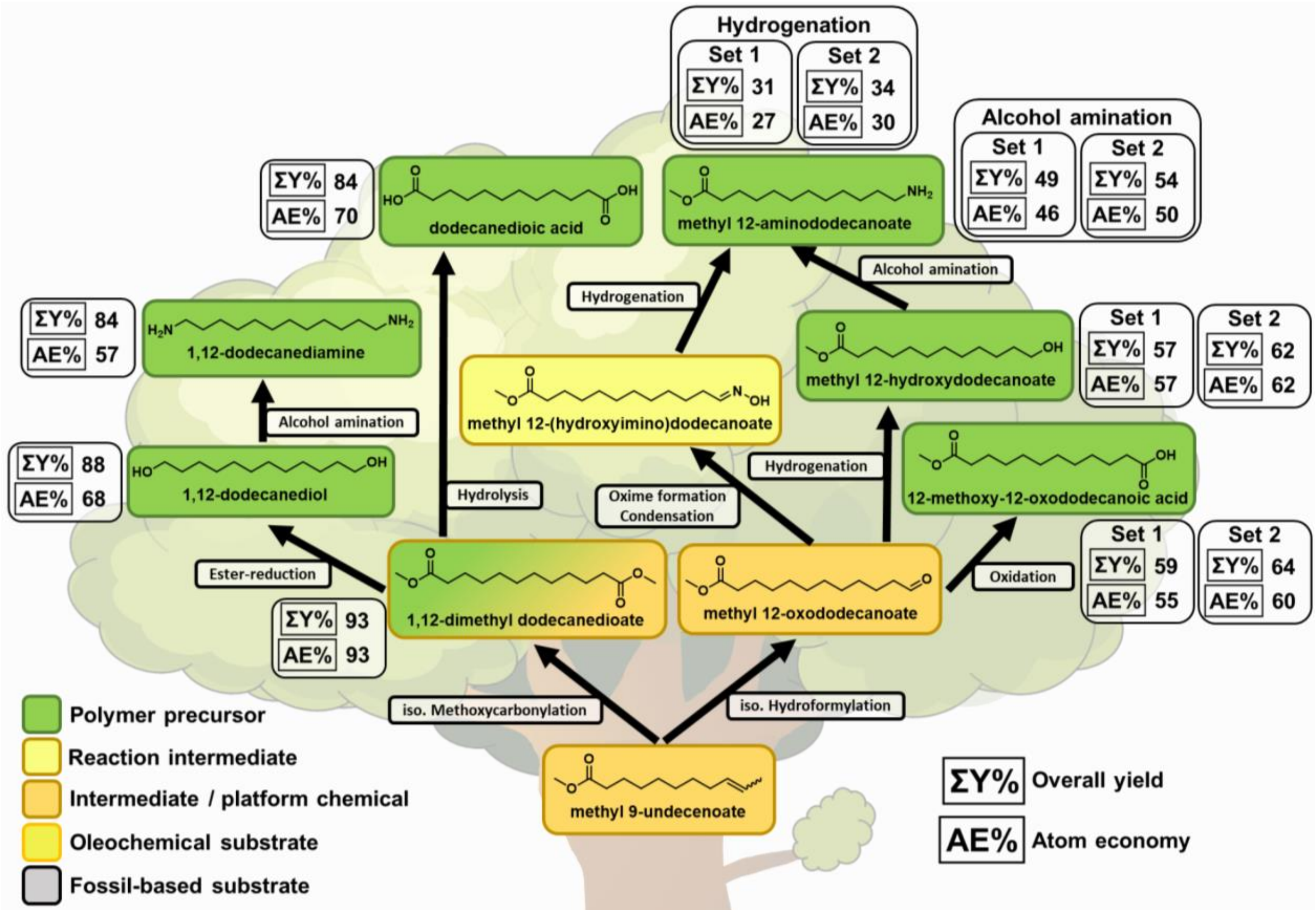

Figure S23: Overall yields (ΣY%) and atom economies (AE%) of the investigated synthetic routes from methyl 9-undecenoate to symmetric and non-symmetric C_{12} polymer precursors. For routes involving isomerising hydroformylation, the values obtained using Sets 1 and 2 are shown separately.

4. References

[1] Apeiron synthesis, "UltraCat," can be found under https://apeiron-synthesis.com/catalysts/ultracat. (accessed: 14.10.2026).
